# High‐Precision and High‐Flux Separation by Rationally Designing the Nanochannels and Surface Nanostructure of Polyamide Nanofiltration Membranes

**DOI:** 10.1002/smsc.202200026

**Published:** 2022-06-11

**Authors:** Han Zheng, Zihao Mou, Yu Jie Lim, Narasimalu Srikanth, Wang Zhang, Sheng Guo, Rong Wang, Kun Zhou

**Affiliations:** ^1^ Environmental Process Modelling Centre Nanyang Environment and Water Research Institute Nanyang Technological University 1 Cleantech Loop Singapore 637141 Singapore; ^2^ Interdisciplinary Graduate Programme Graduate College Nanyang Technological University 61 Nanyang Drive Singapore 637553 Singapore; ^3^ Institute for Advanced Study Chengdu University 2025 Chengluo Avenue Chengdu 610106 P. R. China; ^4^ Singapore Membrane Technology Centre Nanyang Environment and Water Research Institute Nanyang Technological University 1 Cleantech Loop Singapore 637141 Singapore; ^5^ Energy Research Institute @ NTU Nanyang Technological University 1 Cleantech Loop Singapore 637141 Singapore; ^6^ School of Mechanical and Aerospace Engineering Nanyang Technological University 50 Nanyang Avenue Singapore 639798 Singapore; ^7^ College of Materials Science and Engineering Zhejiang University of Technology Hangzhou 310014 P. R. China; ^8^ School of Chemistry and Environmental Engineering Wuhan Institute of Technology Wuhan 430205 P. R. China

**Keywords:** carbon dots, nanofiltration, membrane selectivity, surface nanostructures, thin-film nanocomposites

## Abstract

High‐precision separation with increased water permeability is critical for efficient membrane‐based water treatment processes. To achieve high selectivity toward different targeted species while allowing rapid water transportation, the structure of the membrane polyamide selective layer requires delicate regulation. Herein, an effective approach to systematically expand the pore size of polyamide layers by incorporating ammonium ion‐modified carbon dots (CDs) into the polyamide network is developed. The ammonium ions with different alkyl chain lengths attached to the CDs create nanochannels of different sizes in the network to lower the energy barrier for water transportation while maintaining high selectivity to targeted species. When the alkyl chain length of the ammonium ions reaches eight carbon atoms (i.e., C_8_ ions), the amphiphilic C_8_‐CDs induce the formation of the ridged nanostructure on the membrane surface and hence the increased membrane filtration area. The resultant thin‐film nanocomposite (TFN) membrane, denoted as the TFN‐C_8_‐CDs membrane, demonstrates a higher Na_2_SO_4_ rejection of 98.9% and NaCl/Na_2_SO_4_ selectivity of 83.1 than the pristine polyamide membrane, together with a tripled pure water permeability of 29.0 L m^−2^ h^−1^ bar^−1^. Herein, a viable approach for ingeniously designing the nanochannels and surface nanostructure of polyamide membranes for more efficient filtration processes is provided.

## Graphical abstract

Core–shell‐structured carbon dots grafted with ammonium ions having different alkyl chain lengths are used to systematically expand the nanochannels in the polyamide layer and create nanoridges on the membrane surface for high‐precision and high‐flux nanofiltration.

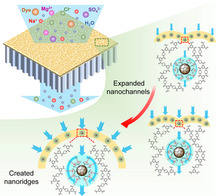

## 1 Introduction

Over the past few decades, membrane technologies have gradually occupied the leading position in various water treatment processes to address the ever‐escalating worldwide water scarcity.^[^ ^1^ ^]^ Thin‐film composite (TFC) membranes comprising a thin yet dense selective layer on top of a mechanically robust porous support are recognized as the state‐of‐the‐art membranes for reverse osmosis (RO), forward osmosis (FO), and nanofiltration (NF) processes because of their high permeability and operational stability.^[^ ^2^ ^]^ For a typical TFC NF membrane, the polyamide active layer formed via an interfacial polymerization (IP) reaction between amine and acyl chloride monomers allows fast transportation of water molecules while effectively rejecting multivalent ions and organic compounds with a molecular weight larger than 200 Da. Although extensive research has been conducted on tuning the surface structure and properties,^[^ ^3^, ^4^ ^]^ reducing the layer thickness,^[^ ^5^, ^6^, ^7^, ^8^ ^]^ loosening the highly crosslinked polymer chains,^[^ ^9^ ^]^ and narrowing the pore size distribution of the selective layer,^[^ ^10^, ^11^ ^]^ the undifferentiated selectivity of the crosslinked polymeric structure based on their exceedingly small pore sizes, which are unfavorable for efficient water transportation, leads to a recurring permeability–selectivity tradeoff.^[^ ^12^ ^]^ In addition to the high energy consumption incurred, the low solute–solute selectivity creates difficulty in the recovery of targeted species from various water sources.^[^ ^13^ ^]^

Recent advances in the synthesis, modification, and assembly of nanomaterials have initiated a research focus on developing high‐performance thin‐film nanocomposite (TFN) NF membranes.^[^ ^14^ ^]^ Typical nanomaterials including Ag,^[^ ^15^ ^]^ TiO_2_,^[^ ^16^ ^]^ and silica nanoparticles,^[^ ^17^ ^]^ biomimetic synthetic nanochannels,^[^ ^18^, ^19^ ^]^ graphene nanosheets,^[^ ^20^ ^]^ carbon nanotubes,^[^ ^21^ ^]^ and metal–organic framework nanocrystals^[^ ^22^, ^23^ ^]^ have been involved in the fabrication of TFN membranes as nanofillers in the active layer. These nanomaterials embedded in the polyamide network create nanochannels at their interface or by their intrinsic pores for more efficient water transportation through the dense selective layer.^[^ ^24^, ^25^ ^]^ Moreover, the hydrophilicity of the membrane surface can be improved by the hydrophilic nanomaterials located in the surface layer to allow rapid water diffusion to the permeate side. The charges of the nanochannels created in the polyamide network can be tuned by functional groups on the nanomaterials to reject the targeted ions or charged solutes via the dielectric exclusion (the solvation energy barrier developed by the change of the dielectric constant inside the nanochannels due to the entry of ions at the solid/solution interface) and Donnan effects (the interaction between the ions and the nanochannels with heterogeneous charge density across the thickness).^[^ ^26^ ^]^ In addition, the presence of the nanomaterials in the IP reaction affects the reaction kinetics, thereby changing the degree of crosslinking, reducing the thickness, and altering the morphology of the polyamide layer for enhanced filtration performances.^[^ ^27^ ^]^ However, nonselective voids or defects can be formed because of the poor size or chemical compatibility between the embedded nanomaterials and the polyamide layer, resulting in drastically declined performance in terms of membrane selectivity. Moreover, nanofillers with low chemical and thermal stability may lose their functions under extreme pH conditions, at elevated temperatures, or after prolonged operation, leading to the degeneration of filtration performances.

Carbon dots (CDs) or carbon quantum dots, with an ultrafine size typically below 10 nm, are gaining increasing attention over the past few years as a promising candidate for photonic, optoelectronic, and electrochemical applications because of their tunable photoluminescence and electronic properties, remarkable physicochemical properties, and low costs.^[^ ^28^, ^29^, ^30^, ^31^ ^]^ Recently, this novel nanomaterial has been further applied in membrane‐based water treatment technologies because of its low toxicity, high chemical stability, abundant surface functional groups (e.g., epoxy, carboxyl, and hydroxyl groups), and excellent chemical compatibility.^[^ ^32^, ^33^, ^34^ ^]^ The superior size and chemical compatibility facilitate the embedment of CDs in the polyamide structure without affecting the integrity of the crosslinked polyamide network. The polar oxygen functional groups on the surface not only minimize severe aggregation of CDs in the aqueous phase for the subsequent IP reaction such that the CDs are evenly distributed in the selective layer to maximize their functionality, but also provide vast potential for further surface modification. Furthermore, CDs with high chemical and thermal stability ensure their intactness under harsh conditions and after long‐term operation. A number of TFN membranes with high flux, retained or improved selectivity, and enhanced antifouling and antichlorination capability have been developed by incorporating CDs with different functional groups.^[^ ^35^, ^36^, ^37^ ^]^ Nonetheless, none of the research works aimed at tailoring the pore sizes and surface nanostructure of polyamide layers using CDs to achieve desired sieving capacity for targeted solutes and higher water flux. Meanwhile, the mechanisms of the altered membrane properties and hence the enhanced filtration performance by the incorporation of CDs require comprehensive investigations.

**Figure 1:**
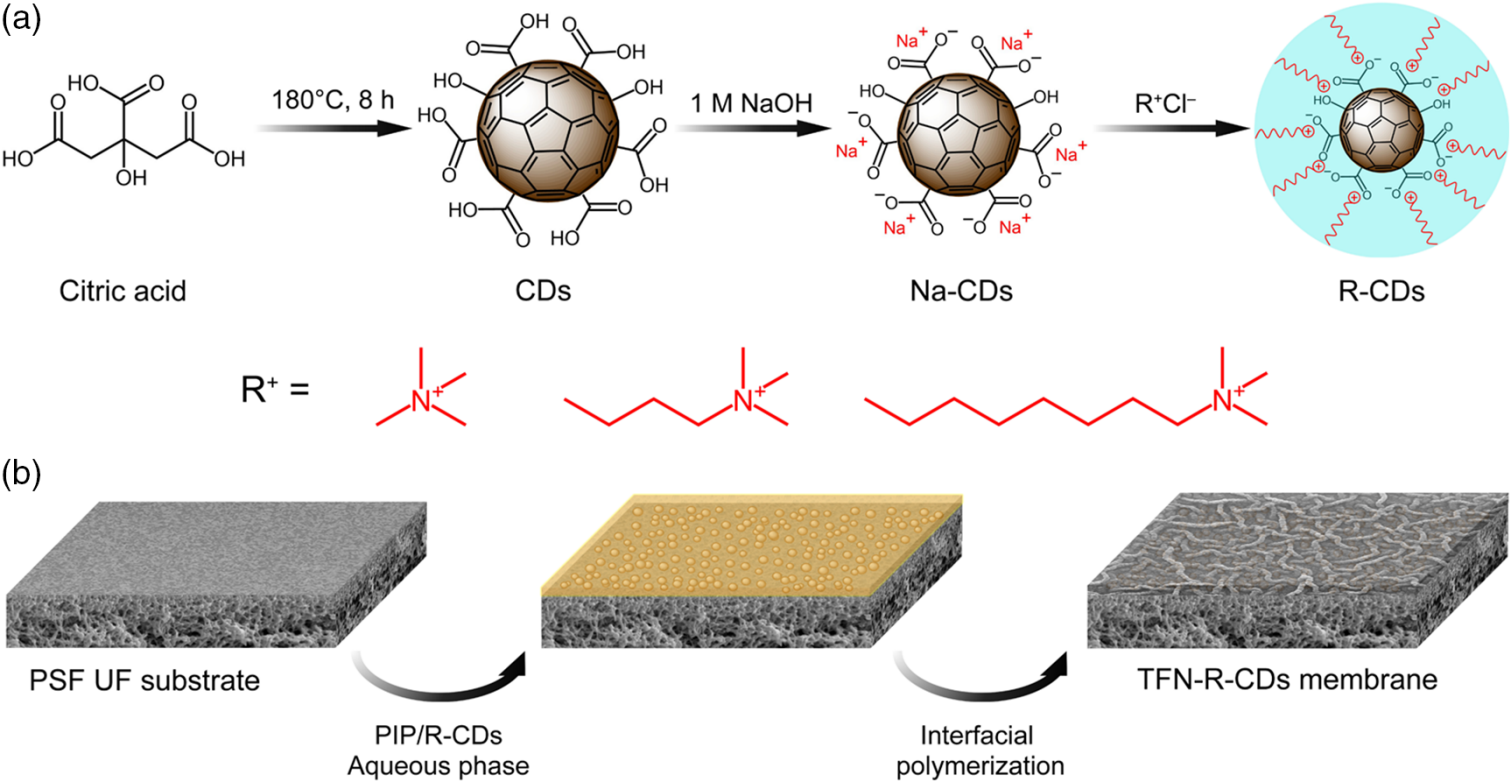
Schematics for a) the synthesis of the core–shell‐structured CDs modified with ammonium ions and b) the preparation of the TFN membranes incorporated with the CDs.

Herein, a series of core–shell‐structured CDs, which comprise a nonpermeable carbonaceous inner core and an outer shell of ammonium ions (i.e., tetramethylammonium, C_1_; butyltrimethylaminium, C_4_; octyltrimethylammonium, C_8_) with different alkyl chain lengths, were developed and denoted as C_1_‐CDs, C_4_‐CDs, and C_8_‐CDs (**Figure** **1**). The ammonium ion‐modified CDs were then incorporated into the TFN membranes (i.e., TFN‐C_1_‐CDs, TFN‐C_4_‐CDs, and TFN‐C_8_‐CD membranes) to systematically expand the pores of the polyamide layer formed on top of a polysulfone (PSF) ultrafiltration (UF) substrate for high‐precision separation and enhanced water permeability. Particularly, the C_8_‐CDs showing amphiphilic properties created a crumpled membrane surface with densely distributed nanosized polyamide strips to further elevate the filtration performance by increasing the effective filtration area of the TFN‐C_8_‐CDs membrane. The unique surface nanostructure and finely expanded nanochannels lead to a tripled pure water permeability value of 29.0 L m^−2^ h^−1^ bar^−1^, while maintaining an even higher NaCl/Na_2_SO_4_ selectivity of 83.1 than those of the TFC control membrane, which is attributed to the enhanced Na_2_SO_4_ rejection rate of 98.9% and the ultralow NaCl rejection rate of 10.3%. The mechanisms of the improved filtration performances and the formation of such a “ridge‐and‐valley” membrane surface morphology by the involvement of C_8_‐CDs were thoroughly investigated. The performance stability of the TFN membranes was demonstrated by varying the operating conditions and through a 14‐day filtration test.

## 2 Results and Discussion

**Figure 2:**
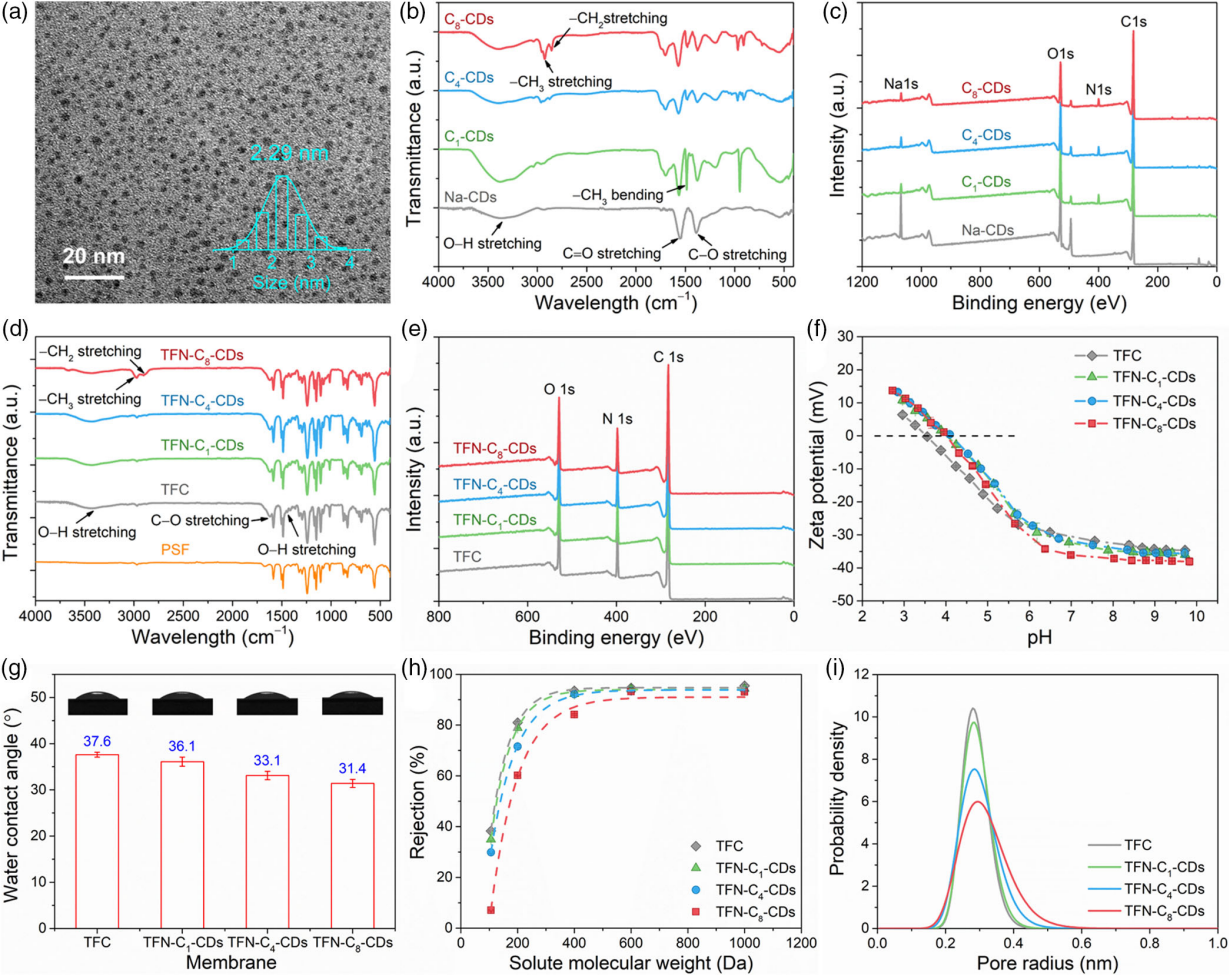
Characterization results of the CDs nanoparticles and membranes: a) the TEM image of C_8_‐CDs; b) the FTIR and c) wide‐scan XPS spectra of the CDs; d) the FTIR spectra, e) the full‐scan XPS spectra, f) the zeta potentials measured in a pH range from 3 to 9.5, g) the water contact angles and the corresponding images of water droplets on the membranes, h) the MWCO results, and i) the pore size distribution of the TFC and TFN‐C_1_‐CDs, TFN‐C_4_‐CDs, and TFN‐C_8_‐CDs membranes. The inset in (a) shows the size distribution of C_8_‐CDs measured using ImageJ software.

The CDs were synthesized by pyrolyzing citric acid under a temperature of 180 °C in air. The Na^+^ ion‐modified CDs (Na‐CDs) were then prepared by dispersing the as‐prepared negatively charged CDs in a 1 M sodium hydroxide (NaOH) solution. Through a cation‐exchange process in water, the ammonium ions (i.e., tetramethylammonium, C_1_; butyltrimethylaminium, C_4_; octyltrimethylammonium, C_8_) replaced the Na^+^ ions adsorbed on the CDs surface to form the core–shell‐structured C_1_‐CDs, C_4_‐CDs, and C_8_‐CDs. It was noted that the addition of ammonium ions with longer alkyl chains (e.g., dodecyltrimethylammonium, C_12_, and hexadecyltrimethylammonium, C_16_) in the dispersion of Na‐CDs induced rapid flocculation, which indicates the hydrophobicity of C_12_‐CDs and C_16_‐CDs due to the long hydrocarbon fragments in the outer shell (Figure S1, Supporting Information). The excellent water dispersibility and the mild alcohol dispersibility suggest the amphiphilic property of C_8_‐CDs. The transmission electron microscopy (TEM) images (Figure S2, Supporting Information) show that the Na‐CDs have an ultrasmall average size of 2.23 nm with narrow size distribution. After the Na^+^ ions were replaced by the ammonium ions, the average size of CDs slightly increased from C_1_‐CDs to C_8_‐CDs (**Figure** **2**a), implying an expanded shell size by the longer alkyl chains. The thermogravimetric analysis (TGA) measurement results (Figure S3, Supporting Information) further verify an increased amount of ammonium ions attached from C_1_‐CDs to C_8_‐CDs, which is reflected by the increased weight percentage loss at the end of the heating processes. The amorphous structure of the CDs with a low carbonization degree is revealed by their X‐ray diffraction (XRD) patterns (Figure S4, Supporting Information) that exhibit broad peaks at around 20° for all the four types of CDs.^[^ ^38^ ^]^

The chemical structure and elemental composition of the CDs were analyzed using Fourier‐transform infrared spectroscopy (FTIR) and X‐ray photoelectron spectroscopy (XPS). In Figure 2b, the broad band at 3380 cm^−1^ (O–H stretching) and the absorption peaks at 1558 (C═O stretching) and 1388 cm^−1^ (carboxyl C–O stretching) signify the formation of CDs.^[^ ^39^ ^]^ Compared with Na‐CDs, the ammonium ion‐modified CDs exhibit a new peak at 1483 cm^−1^, which is ascribed to the bending vibration of –CH_3_ in the ammonium group. The emerging peaks at 2926 and 2856 cm^−1^ in the spectra of C_4_‐CDs and C_8_‐CDs are assigned to the asymmetric and symmetric stretching vibrations of –CH_3_ and –CH_2_ in the ammonium ions, respectively.^[^ ^40^ ^]^

The emergence of N 1*s* peaks in the XPS spectra of C_1_‐CDs, C_4_‐CDs, and C_8_‐CDs further confirms the successful attachment of ammonium ions to the CDs via the ion‐exchange process to form the core–shell‐structured nanoparticles (Figure 2c). Meanwhile, the presence of weak Na 1*s* peaks in the spectra indicates that a small portion of Na^+^ ions (0.71–0.84 wt%, Table S1, Supporting Information) is retained in the ammonium ion‐modified CDs. The incomplete substitution of Na^+^ ions by the excessive amount of ammonium ions is due to the large steric hindrance imposed by the ammonium head groups. Moreover, the C═O, C—O, and C—C/C═C bonds identified by deconvoluting the C1*s* and O1*s* peaks in their respective high‐resolution spectra (Figure S5 and Table S2, Supporting Information) are reminiscences of the formation of hydrophilic CDs with abundant carboxyl and hydroxyl groups.^[^ ^36^ ^]^

The zeta potential measurement conducted on the CDs revealed their negative overall surface charges (Figure S6, Supporting Information). The more negative charge values from Na‐CDs (−8.9 mV) to C_1_‐CDs (−14.1 mV), C_4_‐CDs (−23.7 mV), and C_8_‐CDs (−27.1 mV) can be explained by the smaller number of the positively charged ammonium ions and the retained Na^+^ ions attached to the surface. Furthermore, from Na^+^ to C_1_, C_4_, and C_8_, which have the same charge number, the increased ionic size leads to the decreased charge density and hence the more negative overall surface charges of the corresponding CDs.

A series of characterizations were conducted on the TFN membranes fabricated using aqueous dispersions containing 0.4 wt% (the optimum loading concentration determined based on their NF performances) C_1_‐CDs, C_4_‐CDs, or C_8_‐CDs. In Figure 2d, the FTIR spectra verify the formation of polyamide layers in the TFC and TFN membranes by showing the characteristic peaks of O–H and N–H stretching (3420 cm^−1^),^[^ ^4^ ^]^ C═O stretching (1618 cm^−1^) in the amide bonds,^[^ ^41^ ^]^ and O–H stretching (1444 cm^−1^) in the carboxyl groups originated from the unreacted acyl chlorides of trimesoyl chloride (TMC).^[^ ^42^ ^]^ In the spectrum of the TFN‐C_8_‐CDs membrane, two emerging peaks at 2971 and 2901 cm^−1^ are related to the –CH_3_ and –CH_2_ groups in the ammonium ions attached on C_8_‐CDs, which confirm the embedment of CDs in the polyamide layer. Note that the FTIR technique has a penetration depth of 0.5–1.0 μm, which is much larger than the thickness of the thin active layer.^[^ ^43^ ^]^ Hence the characteristic peak of the ammonium ion‐modified CDs at 1483 cm^−1^ is covered by the signal of the PSF support at 1487 cm^−1^.

The elemental compositions of the membrane surfaces were determined by XPS. In the survey spectra of the polyamide layers, the peaks at 284.8, 399.1, and 531.2 eV correspond to binding energy of C 1*s*, N 1*s*, and O 1*s*, respectively (Figure 2e). Because the analysis depth of XPS is typically smaller than 10 nm, the missing Na 1*s* signal (originated from the incorporated CDs) implies that at least most of the CDs are embedded at a certain distance below the membrane surface.^[^ ^26^ ^]^ Thus, the degrees of crosslinking of the polyamide layers can be directly calculated based on the atomic ratios of O to N obtained from the XPS measurements (**Table** **1**) without considering the interference from the CDs (see the detailed calculation method in Section S1.6, Supporting Information). From the TFC to the TFN‐C_1_‐CDs, TFN‐C_4_‐CDs, and TFN‐C_8_‐CDs membranes, the degree of crosslinking decreases from 69.6% to 64.4%, 63.6%, and 63.0%, indicating a looser polyamide structure.

**Table 1:** Characteristics of the membranes based on XPS analysis and MWCO measurement

Membrane	Surface elemental composition and concentration			Degree of crosslinking [%]	MWCO [Da]	Mean pore radius [nm]
	C 1*s* [%]	N 1*s* [%]	O 1*s* [%]			
TFC	75.07	11.20	13.73	69.6	272	0.286
TFN‐C_1_‐CDs	75.19	10.94	13.88	64.4	293	0.289
TFN‐C_4_‐CDs	75.21	10.89	13.90	63.6	353	0.294
TFN‐C_8_‐CDs	75.20	10.87	13.93	63.0	529	0.309

The deconvoluted high‐resolution XPS spectra for the C 1*s*, N 1*s*, and O 1*s* peaks of the membranes provide additional evidence for the looser structure of the polyamide layers in the TFN membranes (Figure S7, Supporting Information). An increasing amount of O═C–O and N–H bonds, which originate from the unreacted acyl chloride groups in TMC and amine groups in piperazine (PIP),^[^ ^44^ ^]^ respectively, is observed from the TFC to the TFN‐C_1_‐CDs, TFN‐C_4_‐CDs, and TFN‐C_8_‐CDs membranes (Table S3, Supporting Information). The results confirm that the TFC membrane has a higher degree of crosslinking than the TFN membranes.

The membrane surface charges in a pH range of 3–9.5 were measured on an electrokinetic analyzer (Anton Paar SurPASS 3) and the results are plotted in Figure 2f. The more positively charged membrane surfaces of the TFN membranes than that of the TFC membrane in the acidic region (pH < 5) are mainly attributed to the larger number of the unreacted amine groups in the looser polyamide structure. Meanwhile, the more negatively charged membrane surfaces of the TFN membrane in the basic region (pH > 7) are ascribed to the larger number of carboxylic acid groups.^[^ ^45^ ^]^ Note that the anionic CDs are embedded below the polyamide surface layer as revealed by the XPS measurement and hence do not contribute to the negative surface charges of the TFN membranes. With the largest number of the unreacted amine and carboxylic acid groups, the TFN‐C_8_‐CDs membrane exhibits highest hydrophilicity, followed by the TFN‐C_4_‐CDs, TFN‐C_1_‐CDs, and TFC membranes, as indicated by the water contact angle analysis (Figure 2g).

The network structures of the polyamide layers in the TFC and TFN membranes were analyzed by filtrating solutions of uncharged organic species including diethylene glycol (DEG, *M*_r_ = 106 Da), poly(ethylene glycol)‐200 (PEG‐200, *M*_r_ = 190–210 Da), PEG‐400 (*M*_r_ = 380–420 Da), PEG‐600 (*M*_r_ = 570–630 Da), and PEG‐1000 (*M*_r_ = 950–1050 Da). The solute concentrations in the feed and permeate solutions were determined using the total organic carbon analysis. Based on the rejection rates of the membranes toward the organic solutes, the molecular weight cutoff (MWCO, calculated at the point that 90% of the solutes are rejected) and pore size distribution were determined (see the details in Sections 1.7 and 1.8, Supporting Information). In Figure 2h, after the incorporation of C_1_‐CDs, the TFN‐C_1_‐CDs membrane shows a slight increase in the MWCO value (293 Da) when compared with the TFC membrane (272 Da). As the shell size of the embedded CDs expands (i.e., C_4_‐CDs and C_8_‐CDs), the MWCO value further increases to 353 and 529 Da for the TFN‐C_4_‐CDs and TFN‐C_8_‐CDs membranes, respectively.

Accordingly, the voids in the selective layer are enlarged from the TFC to the TFN‐C_1_‐CDs, TFN‐C_4_‐CDs, and TFN‐C_8_‐CDs membranes, resulting in a systematically increased mean effective pore size (radius) from 0.286 to 0.289, 0.294, and 0.309 nm, respectively (Figure 2i). The rightward spreading of the pore size distribution further signifies that the systematic increase in the mean pore size is mainly due to the creation of larger nanosized channels by the ammonium ions with longer alkyl chains attached to the CDs. Note that the introduction of C_1_‐CDs in the polyamide network induces a significant drop in the degree of crosslinking but a marginal change in the pore size, while the further increase in the alkyl chain length effectively expands the pore size but results in an insignificant decrease in the degree of crosslinking for the TFN membranes. The mismatch of the variation trends indicates the negligible contribution of the slightly loosened polyamide structure to the systematical expansion of the pore sizes from the TFN‐C_1_‐CDs to TFN‐C_8_‐CDs membranes.

**Figure 3:**
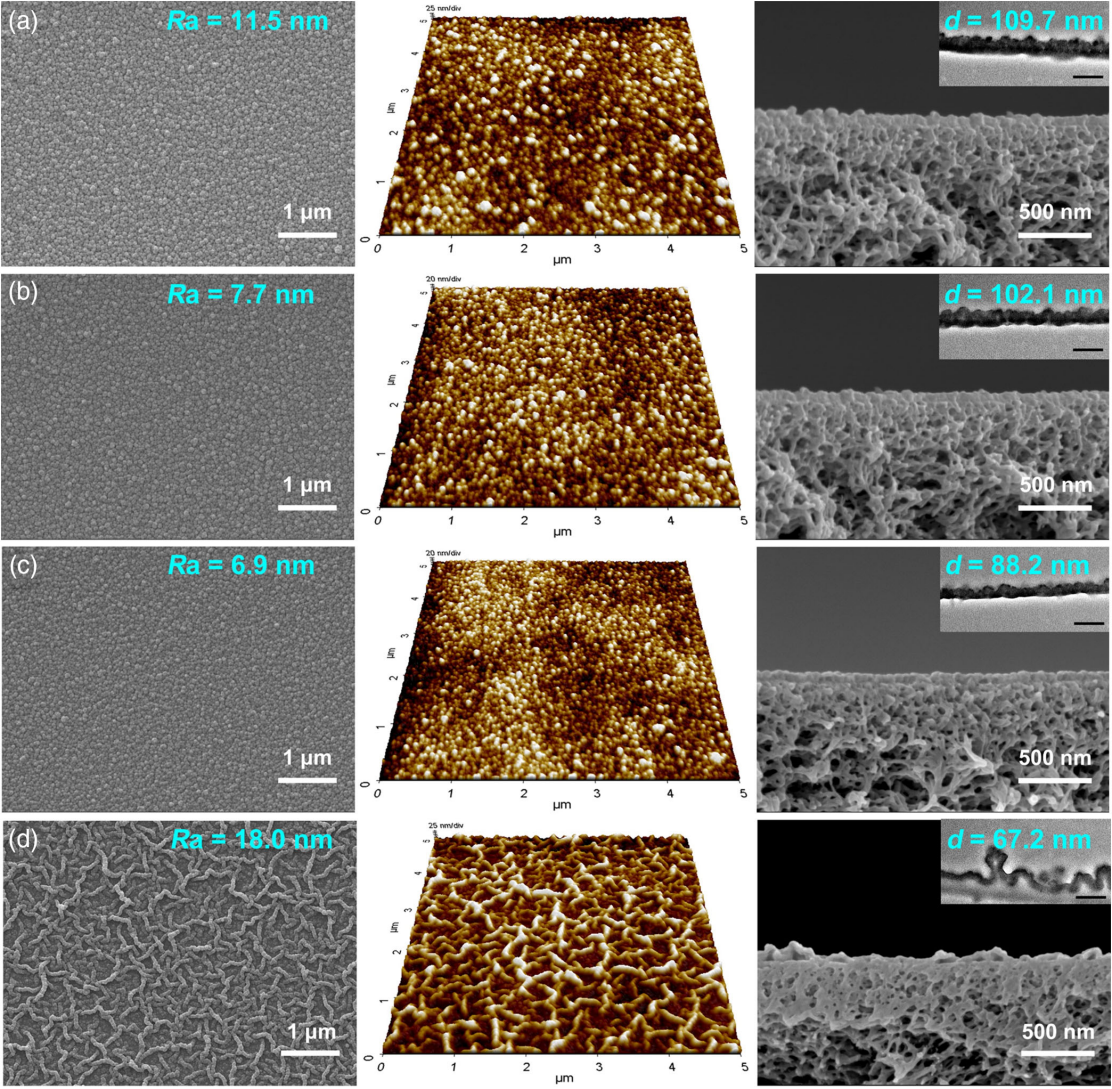
Morphology of the a) TFC, b) TFN‐C_1_‐CDs, c) TFN‐C_4_‐CDs, and d) TFN‐C_8_‐CDs membranes: FESEM (left panels, with the surface roughness values *R*_a_ labeled) and AFM images (middle panels) of the membrane surfaces; FESEM images of the membrane cross sections (right panels). The insets in the right panels display the TEM cross‐sectional images of the polyamide layers and the measured layer thickness *d* (scale bar: 100 nm).

The morphology of the membrane surfaces and cross sections was revealed by field‐emission scanning electron microscopy (FESEM), atomic force microscopy (AFM), and TEM. In **Figure** **3**a, small nodules are densely distributed on the surface of the TFC membrane with an arithmetic average roughness value *R*_a_ of 11.5 nm and a resultant surface area increase of 11.1% (Table S4, Supporting Information), implying an intensive IP reaction between PIP and TMC monomers. It is widely recognized that the diffusion of PIP across the aqueous/organic interface is the rate‐limiting step of the IP reaction.^[^ ^8^ ^]^ After the addition of C_1_‐CDs or C_4_‐CDs in the aqueous phase, the negatively charged nanoparticles attract plenty of cationic PIP molecules on their surfaces to form CDs/PIP complexes. During the IP process, the well‐distributed complexes at the water/hexane interface rapidly react with the TMC monomers while effectively impeding the subsequent diffusion of the PIP molecules to the reaction interface via steric hindrance and electrostatic repulsion.^[^ ^46^, ^47^ ^]^ The combination of the initial electrostatic attraction and the subsequent hindrance and repulsion of C_1_‐CDs and C_4_‐CDs to the PIP molecules resulted in the evenly distributed bulges formed on the smoother surfaces of the TFN‐C_1_‐CDs and TFN‐C_4_‐CDs membranes with smaller *R*_a_ values of 7.7 and 6.9 nm and surface area increases of 9.1% and 7.8%, respectively (Figure 3b,c).

In contrast, a ridge‐and‐valley structure was generated when C_8_‐CDs were involved in the IP reaction, leading to a much larger *R*_a_ value of 18.0 nm and surface area increase of 24.1% (Figure 3d). Because of the amphiphilic properties of the nanoparticles after being modified with C_8_ ammonium ions, mild aggregation of C_8_‐CDs could be incurred on the substrate surface after the excessive aqueous solution was removed by the air knife. The unevenly distributed C_8_‐CDs together with the attracted PIP monomers on their surfaces could readily penetrate into the deeper region of the n‐hexane phase, producing a crumpled active layer surface.^[^ ^10^, ^42^ ^]^

In a comparable manner, the active layer thickness *d* decreases from the TFC membrane (109.7 nm) to the TFN‐C_1_‐CDs (102.1 nm) and TFN‐C_4_‐CDs (88.2 nm) membranes because of the suppressed IP reaction after the introduction of C_1_‐CDs and C_4_‐CDs. The ridge‐and‐valley structure with voids underneath the nanoscale polyamide ridges can be clearly observed in the cross section of the TFN‐C_8_‐CDs membrane and the back surface of the polyamide layer (Figure S9, Supporting Information). The C_8_‐CDs/PIP complexes with a larger hydrated radius further slowed down the IP reaction to achieve the smallest membrane thickness of 67.2 nm.

Prior to the investigation of the NF performances of the TFC and TFN membranes, the optimum loading concentration of CDs in the TFN membranes was determined by dispersing 0.1–0.6 wt% of C_1_‐CDs, C_4_‐CDs, and C_8_‐CDs in the aqueous phase for the IP reaction. The water permeability and Na_2_SO_4_ rejection rates of the TFN membranes prepared are displayed in Figure S10, Supporting Information. On the one hand, the continuously improved water permeability by increasing the loading concentration of the three types of CDs is primarily attributed to the expanded pore sizes after the introduction of the core–shell‐structured nanomaterials. In contrast to the trends of the other two TFN membranes, an upsurge in the water permeability of the TFN‐C_8_‐CDs membrane occurred when the loading concentration was increased from 0.2 to 0.4 wt%, which is caused by the drastically increased membrane surface area after the formation of the crumpled structure (Figure S11 and S12 and Table S5, Supporting Information). Moreover, the larger shell size of C_8_‐CDs than C_4_‐CDs and C_1_‐CDs created wider and longer nanochannels in the polyamide network, whereby the permeability values exhibit an ascending trend from the TFN‐C_1_‐CDs to TFN‐C_4_‐CDs and TFN‐C_8_‐CDs membranes with the same loading concentration of CDs.

On the other hand, defects were likely present in the highly wrinkled polyamide layer when an excessively large concentration of C_8_‐CDs (i.e., larger than 0.4 wt%) was involved in the membrane preparation, resulting in the sharp drop in the Na_2_SO_4_ rejection rate of the TFN‐C_8_‐CDs membrane.^[^ ^48^ ^]^ Slight decreases in the salt rejection rates are also observed for the TFN‐C_1_‐CDs and TFN‐C_4_‐CDs membranes with loading concentrations greater than 0.4 wt%.^[^ ^46^ ^]^ By considering both the permeability and rejection performances, the optimum concentration of 0.4 wt% CDs was used in the preparation of the TFN membranes for all the characterization and filtration tests.

**Figure 4:**
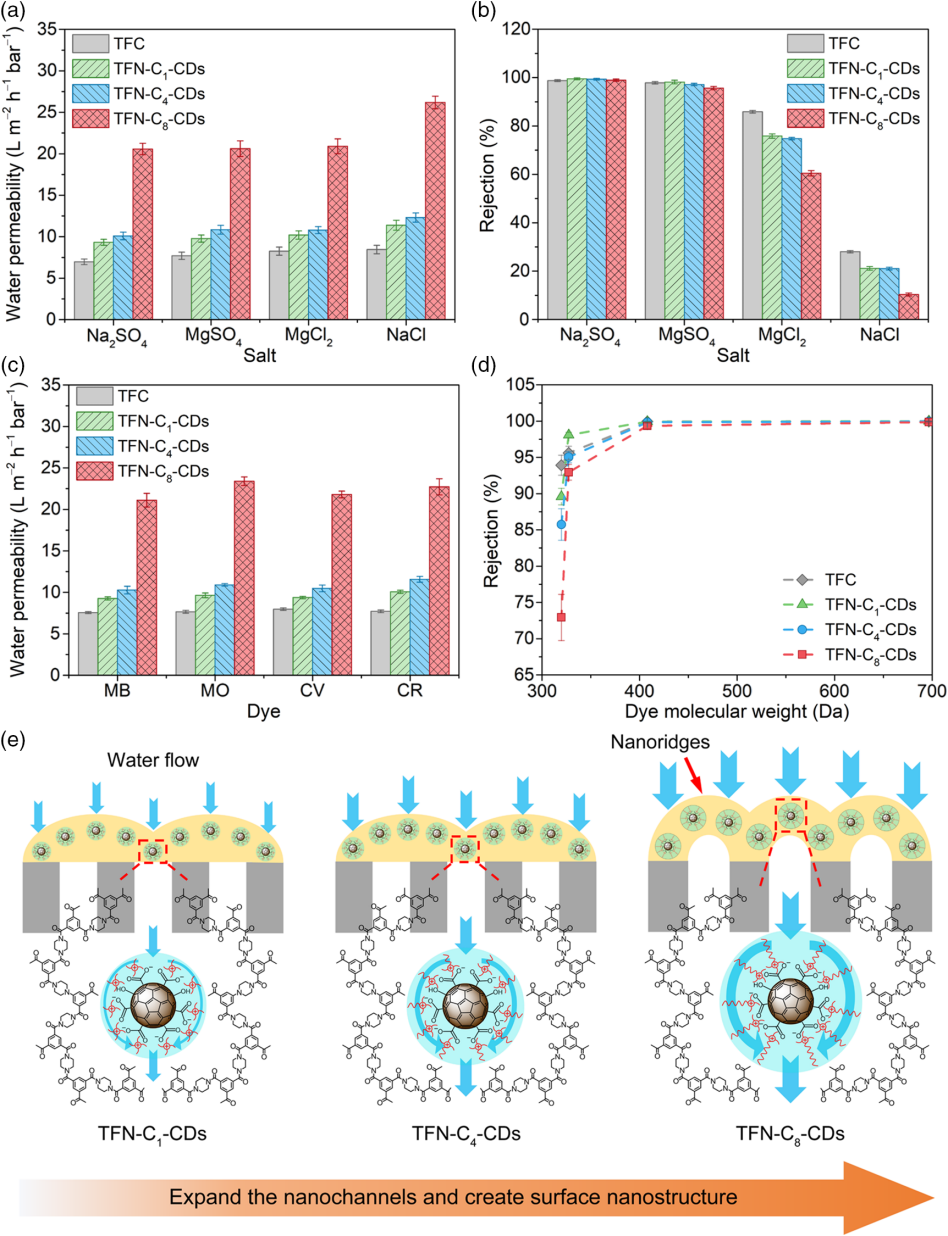
NF performances of the TFC and TFN membranes: a) water permeability and b) rejection rates for treating different salt solutions; c) water permeability and d) rejection rates for treating different dye solutions. The filtration tests were conducted using an aqueous feed solution of 2000 ppm salt or 200 ppm dye under a pressure of 6 bar at 25 °C. e) Schematic illustration of the systematically expanded nanochannels and tailored surface nanostructure by the incorporated core–shell‐structured CDs for the improved NF performances of the TFN membranes.

The NF performances of the TFN membranes were evaluated by treating a group of monovalent and divalent salt ions (i.e., 2000 ppm Na_2_SO_4_, MgSO_4_, MgCl_2_, and NaCl). The TFN‐C_8_‐CDs membrane demonstrates the highest permeability values for all the four sets of filtration, followed by the TFN‐C_4_‐CDs, TFN‐C_1_‐CDs, and TFC membranes (**Figure** **4**a). Particularly, the salt water permeability for the treatment of the Na_2_SO_4_ and MgSO_4_ solutions increases from 7.0 and 7.7 L m^−2^ h^−1^ bar^−1^ for the TFC membrane to 20.6 and 20.7 L m^−2^ h^−1^ bar^−1^ for the TFN‐C_8_‐CDs membrane, respectively. The rejection rates of the TFN membranes toward the divalent salt ions (i.e., Na_2_SO_4_ and MgSO_4_) are comparable with those of the TFC membrane (Figure 4b). Because of the enlarged pore sizes, the rejection to the monovalent ions (i.e., MgCl_2_ and NaCl) is in descending order from the TFC membrane to the TFN‐C_1_‐CDs, TFN‐C_4_‐CDs, and TFN‐C_8_‐CDs membranes. Notably, the TFN‐C_8_‐CDs membrane exhibits an ultralow rejection rate of 10.3% to the NaCl solution and hence a high NaCl/Na_2_SO_4_ selectivity of 83.1. Although the monovalent to divalent ion selectivity will be slightly different when a mixture of NaCl and Na_2_SO_4_ solutions is used as the feed solution,^[^ ^26^ ^]^ the single salt selectivity computed is still of high significance to demonstrate the efficient solute–solute separation of the nanofiltration membranes.^[^ ^49^ ^]^

To further demonstrate the regulated size‐sieving capacity of the TFN membranes based on their finely tuned pore sizes, different dyes including methylene blue (MB), methyl orange (MO), crystal violet (CV), and Congo red (CR) with a wide range of molecular weights (MB: 320 Da; MO: 327 Da; CV: 408 Da; CR: 697 Da) and different charges (positively charged: MB and CV; negatively charged: MO and CR) were used in the filtration test. The rejection performance of the membranes agrees well with the MWCO and pore size distribution results that the TFC membrane exhibits the highest rejection to all the dyes except MO, followed by the TFN‐C_1_‐CDs, TFN‐C_4_‐CDs, and TFN‐C_8_‐CDs membranes (Figure 4d).

The exceptionally high rejections of the TFN membranes toward MO, especially for the TFN‐C_4_‐CDs (353 Da) and TFN‐C_8_‐CDs (529 Da) membranes with MWCO values larger than the molecular weight of MO (327 Da), are probably attributed to the Donnan effects and dielectric exclusion by the negatively charged polyamide network and water channels.^[^ ^50^ ^]^ For the same reason, the TFN‐C_1_‐CDs membrane has a rejection rate slightly lower than 90% against cationic MB molecules, although the MWCO of the membrane (293 Da) is higher than the molecular weight of MB (320 Da). By systematically tuning the pore size of the polyamide layer, the membrane‐sieving capacity and permeability performance can be ingeniously designed to achieve high‐precision separation and high‐flux filtration simultaneously. For instance, the TFN‐C_1_‐CDs and TFN‐C_4_‐CDs membranes demonstrate comparable rejection rates toward MO molecules to the TFC membrane with 26% and 42% increases in the permeate flux, respectively (Figure 4c). Moreover, the TFN‐C_8_‐CDs membrane exhibits ultrahigh selectivity of 99.7% and 99.9% to CV and CR with 2.7‐ and 3.0‐times higher fluxes than the TFC membrane, respectively.

On the basis of the characterization and filtration performance results, the improved water permeability and regulated sieving capacity of the TFN membranes are primarily due to the creation of nanochannels by the alkyl chains attached to the CDs in the polyamide network (Figure 4e). Instead of overcoming a higher energy barrier to diffuse across the highly dense polyamide network, the water molecules can take a supplementary transport pathway through the nanochannels. As the length of the alkyl chains increases from one to eight carbon atoms, the nanochannels are systematically expanded to expedite water transportation while retaining high selectivity toward solutes with sizes just larger than the channel diameter to simultaneously achieve high‐flux filtration and high‐precision separation of targeted species. The unique crumpled polyamide structure of the TFN‐C_8_‐CDs membrane further accelerates the water transportation across the membrane by providing an additional membrane filtration area. Moreover, the reduced membrane thickness via the controlled IP reaction and the hydrophilic surface of the polyamide layer with a declined degree of crosslinking contribute to the enhanced water permeability and altered selectivity of the TFN membranes.

The mechanisms of the formation of the ridged nanostructure were investigated and the filtration performances, particularly the pure water permeability and NaCl/Na_2_SO_4_ ion selectivity, of the TFN‐C_8_‐CDs membrane were further optimized by manipulating the concentrations of PIP (0.5–1.5 wt%) and TMC (0.05–0.25 wt%). Note that the effects of the C_8_‐CDs concentration on the membrane surface morphology have been discussed in Figure S11 and S12, Supporting Information, while the pure water permeability and NaCl/Na_2_SO_4_ selectivity reach an optimum combination when the loading concentration is 0.4 wt% (Figure S13, Supporting Information). Lower pure water permeability and higher NaCl/Na_2_SO_4_ selectivity were obtained when a more concentrated PIP aqueous phase was used, whereas the opposite trends were observed when the TMC concentration was increased (Figure S14, Supporting Information). The results suggest that the formation of a dense polyamide layer embedded with amphiphilic C_8_‐CDs requires not only a high PIP concentration but also a high concentration ratio of PIP to TMC, because an additional PIP molecule is needed to crosslink each pair of the repeating units from two adjacent linear polyamide chains.

The membrane nanostructure was also affected by the PIP and TMC concentrations (Figure S15 and S16, Supporting Information). A higher PIP concentration induces more densely distributed and thicker polyamide nanostrips, which create a greater energy barrier for water and ions transportation. In contrast, a more concentrated TMC phase leads to rougher nanostrips and an increasing number of polyamide nodules (i.e., the secondary or grafted nodules) occupying the valley regions, which are prone to defects formation on the primary nodules and strips.^[^ ^51^ ^]^

Based on the effects of PIP, TMC, and C_8_‐CDs concentrations, the generation of nanoscale ridges is mainly attributed to the heterogeneous distribution of highly concentrated C_8_‐CDs/PIP complexes on the substrate surface. When the concentration of C_8_‐CDs in the aqueous phase reaches a threshold value, mild local aggregation of these amphiphilic nanoparticles may occur on the substrate surface after being dried for the subsequent IP reaction. The negatively charged aggregates that attract a large number of PIP molecules readily diffuse toward and penetrate the water/hexane interface to promote the formation of the nanoscale polyamide strips.^[^ ^23^ ^]^ Moreover, sufficient TMC monomers are required to trigger an intensive IP reaction to hinder the diffusion of subsequent diffusion of PIP and TMC monomers toward the reaction interface to smooth the ridges.

Similar ridge‐and‐valley surface morphologies were obtained when surfactants such as sodium dodecyl sulfate, sodium dodecyl benzene sulfonate, and cetyltrimethylammonium bromide with a hydrophilic moiety and a long hydrocarbon chain were used in the membrane fabrication to enhance the heterogeneous wetting of the porous substrate.^[^ ^10^, ^52^ ^]^ The role of the C_8_ ammonium ions in the creation of the ridged nanostructure and the enhanced filtration performance was examined by varying the concentration ratio of C_8_‐CDs to C_8_ in the membrane preparation. The addition of C_8_ ammonium ions deteriorates the membrane selectivity and slightly improves the pure water permeability (Figure S13 vs. S17, Supporting Information). With the concentration of C_8_‐CDs fixed at 0.4 wt%, the additional C_8_ ions lead to the formation of thicker polyamide ridges on the membrane surface with larger and smoother valley regions (Figure S18, Supporting Information). The repulsion between the free cationic C_8_ ammonium ions and PIP monomers in the aqueous phase impedes the transinterface diffusion of the latter, resulting in an unregulated pore size distribution. In the meantime, a higher concentration of PIP is attracted to the C_8_‐CDs aggregates to produce the thicker ridges, resulting in the undersupply of the monomers in other regions, which corresponds to the smoother valley region.

**Figure 5:**
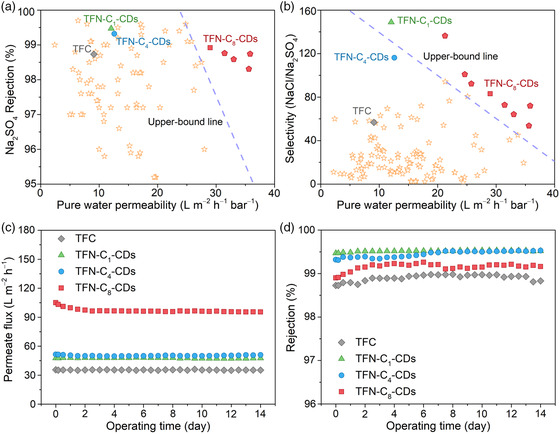
Trade‐off relationship between the pure water permeability and a) Na_2_SO_4_ rejection or b) single salt selectivity (NaCl/Na_2_SO_4_) of the TFC, TFN‐C_1_‐CDs, TFN‐C_4_‐CDs, and TFN‐C_8_‐CDs membranes and other recently NF membranes.^[S5, S7, S14−S80]^ The red square represents the TFN‐C_8_‐CDs membrane prepared using 1.0 wt% PIP, 0.15 wt% TMC, 0.4 wt% C_8_‐CDs, and 0 wt% C_8_, while the red pentagons indicate other high‐performance TFN‐C_8_‐CDs membranes fabricated using different PIP, TMC, C_8_‐CDs, and C_8_ concentrations. Note that the comparison here is just for a brief demonstration as the NF performances are affected by the salt concentration, applied pressure, crossflow velocity, and other testing conditions.^[^ ^5^ ^]^ c,d) Permeate flux and Na_2_SO_4_ rejection of the TFC and TFN membranes under long‐term operation.

The TFN‐C_8_‐CDs membranes fabricated with optimum concentrations of monomers and nanoparticles, together with the TFC, TFN‐C_1_‐CDs, and TFN‐C_4_‐CDs membranes, are compared with commercially available and recently reported lab‐prepared NF membranes for their NF performances including the pure water permeability, divalent ion rejection, and monovalent/divalent ion selectivity in **Figure** **5**a,b. Attributed to the unique intrinsic and surface structure, the TFN‐C_8_‐CDs membranes demonstrate one of the best permeability and selectivity performances. Further modification of the core–shell structure and manipulation of the membrane preparation conditions could extend the application of the CDs‐based TFN membranes to other efficient solute–solute separation processes in textile printing and dyeing, metallurgical and mining, and pharmaceutical industries. Moreover, although comprehensive characterization experiments have been conducted to verify the role of ammonium ion‐modified CDs with different shell sizes in tailoring the pore size and surface nanostructure of the TFN membranes for improved selectivity and permeability, molecular dynamics simulation to be conducted in our future work is critical to obtaining a deep theoretical understanding of the interaction of the nanoparticles with the monomers during the IP process and the intrinsic structure of the resultant polyamide network.

To evaluate the stability of the filtration performances under different operating conditions, the permeate flux and rejection rates (taking Na_2_SO_4_ as an example) of the TFC and TFN membranes were measured by varying the applied pressure, salt concentration, and operating temperature (Figure S19, Supporting Information). The flux values of all the membranes show high linearity under a wide range of pressure from 2 to 10 bar, while the rejection performance is improved when the pressure is increased because the relatively constant salt flux through the membrane is diluted by the enhanced water flux. A higher salt concentration leads to the slightly decreased permeate flux (due to the intensified concentration polarization effect)^[^ ^3^ ^]^ and declining rejection performance (due to the charge screening effect and Donnan exclusion)^[^ ^53^ ^]^ of all the four membranes. The increase in the operating temperature from 25 to 60 °C boosts the permeate flux while slightly reducing the rejection rates of the membranes as a result of the lower water viscosity and expanded nanochannels.^[^ ^2^, ^54^ ^]^

To obtain a more direct view of the filtration stability of the TFN membranes with plenty of subnanoscale pores and/or a unique ridged nanostructure (i.e., the TFN‐C_8_‐CDs membrane) created for rapid water transportation, the permeate flux of the TFN membranes was normalized by that of the TFC membrane under various filtration conditions (Figure S20, Supporting Information). The highly stable permeate flux ratios of all the TFN membranes, despite the minor fluctuations in the permeate flux ratios when solutions of different salts and organic solutes were treated, indicate that the core–shell‐structured CDs and the surrounding polyamide structure remain intact, and the water channels and surface nanostructure created have negligible deformation under a harsh operating environment.

The long‐term stability of the membranes was assessed through a continuous filtration test for 14 days under a pressure of 6 bar. Both the TFN and TFC membranes demonstrate stable flux and Na_2_SO_4_ rejection performances throughout the tests (Figure 5c,d), indicating that the pore structure of the TFN membranes is well maintained, and the embedment of CDs in the polyamide structure does not induce any nonselective defects under prolonged operation. The slight decrease in the permeate flux values of the TFN‐C_8_‐CDs membrane could be due to the compression of the crumpled membrane structure, as the FESEM images reveal that there are plenty of voids between the “ridge” part of the polyamide layer and the substrate.^[^ ^52^ ^]^ Nonetheless, a plateau in the trend of water flux is reached after 2 days, and a high flux value (95.4 L m^−2^ h^−1^) was still retained at the end of the 14 days as compared with the initial value of 105.1 L m^−2^ h^−1^.

## 3 Conclusion

In this work, an effective approach to systematically tune the pore size of polyamide NF membranes for high‐precision separation and high‐flux filtration was developed by incorporating ammonium ion‐modified core–shell‐structured CDs into the selective layer. The cationic ammonium ions with different alkyl chain lengths as the shell attached to the negatively charged carbonaceous core of CDs created nanochannels in the polyamide layer to regulate the pore size. Through the subtle design of the membrane polyamide network for the precise separation of different solutes, the pore size can be finely tuned to be just smaller than the hydrated radius of the target species to maintain high selectivity while allowing faster water transportation.

When the alkyl chain length of the ammonium ions on the CDs surface reached a critical value (i.e., eight carbon atoms), the ammonium ion‐modified CDs (i.e., C_8_‐CDs) showing amphiphilic properties induced the formation of a ridged nanostructure on the membrane surface, which increases the membrane filtration area and enhances the permeability. The emergence of the nanoscale polyamide strips is ascribed to the heterogeneous distribution of highly concentrated C_8_‐CDs/PIP complexes on the substrate due to the mild local aggregation of the amphiphilic C_8_‐CDs after surface drying for the subsequent IP reaction. The nanostructure of the polyamide layer can be further tailored by manipulating the concentrations of PIP and TMC monomers, C_8_‐CDs nanoparticles, and C_8_ ammonium ions used for the IP reaction.

By virtue of the enlarged pore size and the increased filtration area, the TFN‐C_8_‐CDs membrane achieved a tripled pure water permeability value of 29.0 L m^−2^ h^−1^ bar^−1^ and a higher NaCl/Na_2_SO_4_ ion selectivity of 83.1 than the TFC membrane. Moreover, all the TFN membranes demonstrated sustained long‐term filtration performance and high operational stability under a wide range of applied pressures, salt concentrations, and operating temperatures. This work contributes to the fit‐for‐purpose design of the membrane nanochannels and surface nanostructure through the development of core–shell‐structured nanofillers for more efficient membrane separation processes.

## Conflict of Interest

The authors declare no conflict of interest.

## Supporting information

Supplementary Material

## Data Availability

The data that support the findings of this study are available on request from the corresponding author. The data are not publicly available due to privacy or ethical restrictions.

## References

[1] R. M. DuChanois , C. J. Porter , C. Violet , R. Verduzco , M. Elimelech , Adv. Mater. 2021, 33, 2101312. 10.1002/adma.202101312 34396602

[2] J. R. Werber , C. O. Osuji , M. Elimelech , Nat. Rev. Mater. 2016, 1, 16018.

[3] H. Peng , W. H. Zhang , W. S. Hung , N. Wang , J. Sun , K. R. Lee , Q. F. An , C. M. Liu , Q. Zhao , Adv. Mater. 2020, 32, 2001383. 10.1002/adma.202001383 32350974

[4] Z. Tan , S. Chen , X. Peng , L. Zhang , C. Gao , Science 2018, 360, 518. 29724951 10.1126/science.aar6308

[5] P. Sarkar , S. Modak , S. Karan , Adv. Funct. Mater. 2021, 31, 2007054.

[6] M. R. Chowdhury , J. Steffes , B. D. Huey , J. R. McCutcheon , Science 2018, 361, 682. 30115806 10.1126/science.aar2122

[7] Z. Jiang , S. Karan , A. G. Livingston , Adv. Mater. 2018, 30, 1705973. 10.1002/adma.201705973 29484724

[8] S. Karan , Z. Jiang , A. G. Livingston , Science 2015, 348, 1347. 26089512 10.1126/science.aaa5058

[9] Y. Zhao , X. Tong , Y. Chen , Environ. Sci. Technol. 2021, 55, 3352. 33596060 10.1021/acs.est.0c08101

[10] Y. Liang , Y. Zhu , C. Liu , K. R. Lee , W. S. Hung , Z. Wang , Y. Li , M. Elimelech , J. Jin , S. Lin , Nat. Commun. 2020, 11, 2015. 32332724 10.1038/s41467-020-15771-2 PMC7181833

[11] J. Liu , D. Hua , Y. Zhang , S. Japip , T. S. Chung , Adv. Mater. 2018, 30, 1705933. 10.1002/adma.201705933 29380439

[12] H. B. Park , J. Kamcev , L. M. Robeson , M. Elimelech , B. D. Freeman , Science 2017, 356, 1137. 10.1126/science.aab0530 28619885

[13] R. Epsztein , R. M. DuChanois , C. L. Ritt , A. Noy , M. Elimelech , Nat. Nanotechnol. 2020, 15, 426. 32533116 10.1038/s41565-020-0713-6

[14] Z. Yang , P.‐F. Sun , X. Li , B. Gan , L. Wang , X. Song , H.‐D. Park , C. Y. Tang , Environ. Sci. Technol. 2020, 54, 15563. 33213143 10.1021/acs.est.0c05377

[15] Z. Yang , H. Guo , Z.‐K. Yao , Y. Mei , C. Y. Tang , Environ. Sci. Technol. 2019, 53, 5301. 30973224 10.1021/acs.est.9b00473

[16] M. I. Baig , P. G. Ingole , J.‐D. Jeon , S. U. Hong , W. K. Choi , H. K. Lee , Chem. Eng. J. 2019, 373, 1190.

[17] H. Shen , S. Wang , H. Xu , Y. Zhou , C. Gao , J. Membr. Sci. 2018, 565, 145.

[18] M. Di Vincenzo , A. Tiraferri , V.‐E. Musteata , S. Chisca , R. Sougrat , L.‐B. Huang , S. P. Nunes , M. Barboiu , Nat. Nanotechnol. 2021, 16, 190. 33169009 10.1038/s41565-020-00796-x

[19] Y. J. Lim , K. Goh , G. S. Lai , C. Y. Ng , J. Torres , R. Wang , J. Membr. Sci. 2021, 628, 119276.

[20] G. Lai , W. Lau , P. Goh , A. Ismail , Y. Tan , C. Chong , R. Krause-Rehberg , S. Awad , Chem. Eng. J. 2018, 344, 524.

[21] S. Gao , Y. Zhu , Y. Gong , Z. Wang , W. Fang , J. Jin , ACS Nano 2019, 13, 5278. 31017384 10.1021/acsnano.8b09761

[22] F. Wang , T. Zheng , P. Wang , M. Chen , Z. Wang , H. Jiang , J. Ma , Environ. Sci. Technol. 2021, 55, 5324. 33728905 10.1021/acs.est.0c07122

[23] J. Zhu , J. Hou , S. Yuan , Y. Zhao , Y. Li , R. Zhang , M. Tian , J. Li , J. Wang , B. Van der Bruggen , J. Mater. Chem. A 2019, 7, 16313.

[24] B. Li , S. Japip , T.‐S. Chung , Nat. Commun. 2020, 11, 1198. 32139689 10.1038/s41467-020-15070-w PMC7057969

[25] Y. J. Lim , K. Goh , M. Kurihara , R. Wang , J. Membr. Sci. 2021, 629, 119292.

[26] Z. L. Qiu , L. F. Fang , Y. J. Shen , W. H. Yu , B. K. Zhu , C. Helix-Nielsen , W. Zhang , ACS Nano 2021, 15, 7522. 33779134 10.1021/acsnano.1c00936

[27] Z. Yang , F. Wang , H. Guo , L. E. Peng , X.‐H. Ma , X.‐X. Song , Z. Wang , C. Y. Tang , Environ. Sci. Technol. 2020, 54, 11611. 32786553 10.1021/acs.est.0c03589

[28] K. Nekoueian , M. Amiri , M. Sillanpää , F. Marken , R. Boukherroub , S. Szunerits , Chem. Soc. Rev. 2019, 48, 4281. 31215906 10.1039/c8cs00445e

[29] F. Yuan , T. Yuan , L. Sui , Z. Wang , Z. Xi , Y. Li , X. Li , L. Fan , Z. A. Tan , A. Chen , Nat. Commun. 2018, 9, 2249. 29884873 10.1038/s41467-018-04635-5 PMC5993800

[30] W. Li , Y. Liu , M. Wu , X. Feng , S. A. Redfern , Y. Shang , X. Yong , T. Feng , K. Wu , Z. Liu , Adv. Mater. 2018, 30, 1800676. 10.1002/adma.201800676 29920795

[31] L. Wang , W. Li , L. Yin , Y. Liu , H. Guo , J. Lai , Y. Han , G. Li , M. Li , J. Zhang , Sci. Adv. 2020, 6, eabb6772. 33008913 10.1126/sciadv.abb6772 PMC7852397

[32] C. Long , Z. Jiang , J. Shangguan , T. Qing , P. Zhang , B. Feng , Chem. Eng. J. 2021, 406, 126848.

[33] D. L. Zhao , T.‐S. Chung , Water Res. 2018, 147, 43. 30296608 10.1016/j.watres.2018.09.040

[34] S. Y. Lim , W. Shen , Z. Gao , Chem. Soc. Rev. 2015, 44, 362. 25316556 10.1039/c4cs00269e

[35] H. Zheng , Z. Mou , K. Zhou , ACS Appl. Mater. Interfaces 2020, 12, 53215. 33185418 10.1021/acsami.0c13386

[36] X. Song , Q. Zhou , T. Zhang , H. Xu , Z. Wang , J. Mater. Chem. A 2016, 4, 16896.

[37] D. L. Zhao , S. Das , T.‐S. Chung , Environ. Sci. Technol. 2017, 51, 14016. 29161033 10.1021/acs.est.7b04190

[38] Q. Shen , Y. Lin , Y. Kawabata , Y. Jia , P. Zhang , N. Akther , K. Guan , T. Yoshioka , H. Shon , H. Matsuyama , ACS Appl. Mater. Interfaces 2020, 12, 38662. 32693571 10.1021/acsami.0c10301

[39] W. Gai , D. L. Zhao , T.‐S. Chung , Water Res. 2019, 154, 54. 30771707 10.1016/j.watres.2019.01.043

[40] L. Wang , A. Wang , J. Hazard. Mater. 2008, 160, 173. 18400385 10.1016/j.jhazmat.2008.02.104

[41] X. Cheng , Y. Qin , Y. Ye , X. Chen , K. Wang , Y. Zhang , A. Figoli , E. Drioli , Chem. Eng. J. 2021, 417, 127976.

[42] L. Bai , Y. Liu , N. Bossa , A. Ding , N. Ren , G. Li , H. Liang , M. R. Wiesner , Environ. Sci. Technol. 2018, 52, 11178. 30175584 10.1021/acs.est.8b04102

[43] M. Bass , V. Freger , J. Membr. Sci. 2015, 492, 348.

[44] X. Zhu , X. Cheng , X. Luo , Y. Liu , D. Xu , X. Tang , Z. Gan , L. Yang , G. Li , H. Liang , Environ. Sci. Technol. 2020, 54, 6365. 32324400 10.1021/acs.est.9b06779

[45] B. Yuan , S. Zhao , P. Hu , J. Cui , Q. J. Niu , Nat. Commun. 2020, 11, 6102. 33257695 10.1038/s41467-020-19809-3 PMC7705655

[46] C. Zhang , K. Wei , W. Zhang , Y. Bai , Y. Sun , J. Gu , ACS Appl. Mater. Interfaces 2017, 9, 11082. 28244726 10.1021/acsami.6b12826

[47] H. Sun , P. Wu , J. Membr. Sci. 2018, 564, 394.

[48] Z. Gu , P. Li , X. Gao , Y. Qin , Y. Pan , Y. Zhu , S. Yu , Q. Xia , Y. Liu , D. Zhao , J. Membr. Sci. 2021, 625, 119144.

[49] Y. Lin , X. Yao , Q. Shen , T. Ueda , Y. Kawabata , J. Segawa , K. Guan , T. Istirokhatun , Q. Song , T. Yoshioka , H. Matsuyama , Nano Lett. 2021, 21, 6525. 34339209 10.1021/acs.nanolett.1c01711

[50] Y. Zhu , H. Zhu , G. Li , Z. Mai , Y. Gu , J. Nanopart. Res. 2019, 21, 217.

[51] X. Song , B. Gan , S. Qi , H. Guo , C. Y. Tang , Y. Zhou , C. Gao , Environ. Sci. Technol. 2020, 54, 3559. 32101410 10.1021/acs.est.9b05892

[52] C. Jiang , L. Tian , Z. Zhai , Y. Shen , W. Dong , M. He , Y. Hou , Q. J. Niu , J. Membr. Sci. 2019, 589, 117244.

[53] Y. L. Ji , B. X. Gu , S. J. Xie , M. J. Yin , W. J. Qian , Q. Zhao , W. S. Hung , K. R. Lee , Y. Zhou , Q. F. An , Adv. Mater. 2021, 33, 2102292. 10.1002/adma.202102292 34346108

[54] K. Tiwari , P. Sarkar , S. Modak , H. Singh , S. K. Pramanik , S. Karan , A. Das , Adv. Mater. 2020, 32, 1905621. 10.1002/adma.201905621 31951297

