# Supplementary material for: High‐Precision and High‐Flux Separation by Rationally Designing the Nanochannels and Surface Nanostructure of Polyamide Nanofiltration Membranes
Source: Small Sci. 2022 Jun 11;2(7):2200026. doi: 10.1002/smsc.202200026 (PMC11935859; doi:10.1002/smsc.202200026)
Supplement: Supplementary file 1 — Supplementary Material [file SMSC-2-2200026-s001.pdf]

Supporting Information

High-Precision and High-Flux Separation by Rationally Designing the Nanochannels and Surface Nanostructure of Polyamide Nanofiltration Membranes

Han Zheng, Zihao Mou, Yu Jie Lim, Narasimalu Srikanth, Wang Zhang, Sheng Guo*, Rong Wang, and Kun Zhou**

H. Zheng, Dr. S. Guo, Prof. K. Zhou
Environmental Process Modelling Centre, Nanyang Environment and Water Research Institute, Nanyang Technological University, 1 Cleantech Loop, Singapore 637141, Singapore
H. Zheng, Y. J. Lim
Interdisciplinary Graduate Programme, Graduate College, Nanyang Technological University, 61 Nanyang Drive, Singapore 637553, Singapore
Dr. Z. Mou
Institute for Advanced Study, Chengdu University, 2025 Chengluo Avenue, Chengdu 610106, P. R. China
Y. J. Lim, Prof. R. Wang
Singapore Membrane Technology Centre, Nanyang Environment and Water Research Institute, Nanyang Technological University, 1 Cleantech Loop, Singapore 637141, Singapore
Dr. Srikanth Narasimalu
Energy Research Institute @ NTU, Nanyang Technological University, 1 Cleantech Loop, Singapore 637141, Singapore
Dr. W. Zhang, Prof. K. Zhou
School of Mechanical and Aerospace Engineering
Nanyang Technological University
50 Nanyang Avenue, Singapore 639798, Singapore
Prof. W. Zhang
College of Materials Science and Engineering, Zhejiang University of Technology, Hangzhou 310014, P. R. China
Prof. S. Guo
School of Chemistry and Environmental Engineering, Wuhan Institute of Technology, Wuhan 430205, P. R. China
E-mail: kzhou@ntu.edu.sg; zhangwang@zjut.edu.cn; guoshengwit@163.com

1. Experimental section

1.1 Materials and chemicals

The polysulfone (PSF) ultrafiltration (UF) membranes were purchased from Guochu Technology, China. Citric acid (CA, ≥ 99.5%), piperazine (PIP, 99%), 1,3,5-Benzenetricarbonyl trichloride (TMC, 98%), n-Hexane (≥ 99%), sodium hydroxide (NaOH, ≥ 98%), sodium sulfate (Na_2SO_4, ≥ 99%), magnesium sulfate ($MgSO_4$, ≥ 97%), sodium chloride (NaCl, ≥ 99%), magnesium chloride ($MgCl_2$, ≥ 98%), potassium chloride (KCl, ≥ 99%), diethylene glycol (DEG, 99%), poly(ethylene glycol)-200 (PEG-200, M_r = 190–210 Da, BioUltra), PEG-400 (M_r = 380–420 Da, BioUltra), PEG-600 (M_r = 570–630 Da, BioUltra), PEG-1000 (M_r = 950–1050 Da, BioUltra), methylene blue (MB, ≥95%), methyl orange (MO, 85%), crystal violet (CV, ≥ 90.0%), and Congo red (CR, ≥97.0%) were obtained from Sigma-Aldrich, Singapore. Tetramethylammonium chloride (TMAC, analytical pure), butyltrimethylaminium chloride (BTMAC, 98%), and octyltrimethylammonium chloride (OTMAC, 99%) were purchased from Macklin, China. All chemicals were used without further purification. Milli-Q deionized (DI) water (18.3 MΩ cm) was used in the preparation of aqueous solutions for all the experiments.

1.2 Synthesis of carbon dots (CDs)

The ammonium ion–modified CDs were synthesized *via* a two-step approach. In the first step, 2 g of CA was heated at 180 °C for 8 h in a 5 mL beaker with a glass cover. The reddish-brown product was dispersed in sufficient amount of 1 M NaOH solution to form Na^+-functionalized CDs (Na-CDs) and dialyzed for at least five days to remove the unreacted precursors and excessive Na^+ ions. In the second step, 10 mL of 1 mmol/mL TMAC, BTMAC, and OTMAC aqueous solution was dropwise added into the water dispersion of 0.6 g Na-CDs, respectively. The ammonium cations (*i.e.*, tetramethylammonium, C_1;

butyltrimethylaminium, C_4, and octyltrimethylammonium, C_8) were attached to the negatively charged surface of CDs *via* the cation-exchange process to form C_1-CDs, C_4-CDs, and C_8-CDs. The dispersion was then dialyzed for at least five days to remove the substituted Na^+ ions and excessive ammonium salts. The final product was freeze-dried for further use. It was noted that the CDs modified with ammonium salts with longer alkyl chains, *e.g.*, dodecyltrimethylammonium chloride (DTMAC) and hexadecyltrimethylammonium chloride (HTMAC), were not dispersible in water (Figure S1).

1.3 Fabrication of membranes

The CDs-incorporated thin-film nanocomposite (TFN) were fabricated *via* a standard interfacial polymerization (IP) reaction. The PSF UF substrate was first immersed in the aqueous phase containing 1.0 wt % PIP and a specific content of CDs for 2 min. The UF substrate was then taken out, and the excess aqueous solution on the active surface was removed by an air knife. Afterwards, the substrate was immersed in the organic phase (n-hexane) containing 0.15 wt % TMC for 1 min to form a dense polyamide layer on top of the substrate active surface. The IP reaction was quenched by dipping the as-formed TFN membrane into the pure n-hexane solution. Subsequently, the membrane was cured in an oven at 60 °C for 15 min. The TFN membranes incorporated with C_1-CDs, C_4-CDs, and C_8-CDs were denoted by TFN-C_1-CDs, TFN-C_4-CDs, and TFN-C_8-CDs, respectively. The thin-film composite (TFC) membrane was prepared in an analogous manner without adding CDs in the aqueous phase. All the membranes were stored in DI water at 4 °C for further use.

1.4 Characterization

All the nanomaterial and membrane samples were freeze-dried before the characterization and analyses. The thermogravimetric analysis (TGA) curves of the CDs and

ammonium salts were recorded on a thermogravimetric analyzer from 25 °C to 700 °C at a heating rate of 5 °C min^{-1} under inert (Ar) atmosphere. The X-ray diffraction (XRD) measurement was conducted on an X-ray diffractometer (Bruker D8 Advance, Bruker Corp., U.S.) using Cu-Kα as the radiation source with a 2θ scanned range of 5° to 80° at a step rate of 10° min^{-1}. Fourier transform infrared spectroscopy (FTIR) spectra with a scanned range of 400–4000 cm^{-1} were collected from a SHIMADZU IRPrestige 21 spectrometer. The elemental composition and concentration of CDs and membranes were obtained *via* X-ray photoelectron spectroscopy (XPS) on an AXIS Supra analysis instrument. Transmission electron microscopy (TEM) was performed on a JEOL JEM-1400Plus instrument at 120 kV to analyze the size distribution of CDs and membrane cross-sections. Field emission scanning electron microscopy (FE-SEM) was conducted on a JEOL JSM-7600F scanning electron microscope to capture the surface morphologies and cross-sections of membranes. The membrane surface roughness was measured by atomic force microscopy (AFM) on a Park NX10 atomic force microscope. The water contact angles (WCAs) of membranes and the water droplet profiles were collected on a DataPhysics OCA 15EC goniometer. Zetasizer Nano ZS equipment was used to determine the zeta potential values of CDs in pH neutral aqueous media, while an Anton Paar SurPASS 3 analyzer was used to measure the zeta potential values of membranes in a pH range of 3 to 9.5 with 0.01 M KCl as the electrolyte solution.

1.5 Membrane performance tests

The membrane filtration tests were performed on a bench-scale cross-flow filtration setup. The cross-flow cell (CF042D, Sterlitech, U.S.A.) has an effective membrane area of 42 cm^2 while the cross-flow velocity was set at 60 L h^{-1}. The filtration system was stabilized under 6 bar at 25 °C (unless specified) for 1 h before the results were recorded. To evaluate

the rejection performance of the membranes, aqueous solutions comprising 2000 ppm inorganic salts (Na_2SO_4, $MgSO_4$, $MgCl_2$, and NaCl) were employed as the feed solution.

The permeate flux J_p (L m^{-2} h^{-1}) was calculated as

$$J_{\mathrm{p}} = \frac{\Delta V}{A \Delta t} \tag{S1}$$

where ΔV (L) is the water or permeate volume, A (m^2) is the effective membrane area, and Δt (h) is the filtration time. The salt or pure water permeability WP (L m^{-2} h^{-1} bar^{-1}) was given as

$$\mathrm{WP} = \frac{\Delta V}{A \Delta t \Delta P} \tag{S2}$$

where ΔP (bar) is the pressure difference between the feed and permeate side.

The rejection rate R of the membranes was determined by

$$R = \left(1 - \frac{C_{\mathrm{p}}}{C_{\mathrm{f}}}\right) \times 100\% \tag{S3}$$

where C_p (ppm) and C_f (ppm) denote the solute concentrations of permeate and feed solutions, respectively. The salt concentrations of the feed and permeate solutions were calculated on the basis of their electric conductivity recorded on a conductivity meter (Orion™ Versa Star Pro™, Thermo Scientific).

The NaCl to Na_2SO_4 ion selectivity $S_{\mathrm{NaCl/Na_2SO_4}}$ was calculated by

$$S_{\mathrm{NaCl/Na_2SO_4}} = \frac{1 - R_{\mathrm{NaCl}}}{1 - R_{\mathrm{Na_2SO_4}}} \tag{S4}$$

where R_{NaCl} and $R_{\mathrm{Na_2SO_4}}$ are the salt rejection rates of the membranes to NaCl and Na_2SO_4, respectively.

1.6 Calculation of the degree of crosslinking of the polyamide layers

Since the CDs modified with ammonium ions did not participate in the IP reaction, the degree of crosslinking of the poly(piperazine-amide) structure can be calculated based on the

O/N elemental ratios measured by XPS. The linear and crosslinked portions of the polyamide structure form by PIP and TMC has the empirical formulas of $(C_{13}H_{12}N_2O_4)_n$ and $(C_{15}H_{15}N_3O_3)_m$, respectively, as shown below.

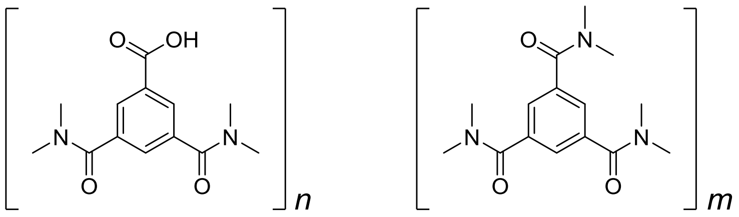

The degree of cross-linking (*D*) can be derived from the different O/N ratios for the linear portion *n* (4/2) and the cross-linked portion *m* (3/3) by

$$\frac{O}{N} = \frac{4n + 3m}{2n + 3m} \tag{S5}$$

$$D = \frac{m}{m + n} \times 100\% \tag{S6}$$

A fully crosslinked membrane (D = 1) has an O/N ratio of 1, while a fully uncrosslinked membrane (D = 0) has an O/N ratio of 2.

Note that the XPS results of the membranes revealed that there was no or negligible amount of Na^+ ions detected in the surface layer, given that there was still a small amount of Na remaining on the surface of the CDs after the modification with ammonium ions, implying that the CDs were embedded below the surface layer and did not contribute to the atomic percentages of the O and N elements in the surface layer of the polyamide structure.

1.7 Determination of the molecular weight cutoff (MWCO) of the TFC and TFN membranes

The MWCO measurement was conducted by filtering aqueous solution containing uncharged organic molecules with different molecular weights, including DEG, PEG-200, PEG-400, PEG-600, and PEG-1000. The concentration of each organic solutes in the feed solution was fixed at 200 ppm and the pressure applied at the feed side was set at 6 bar. The filtration system was stabilized by compressing all the membranes under a pressure of 6 bar

for 1 h before the permeate was collected. The concentrations of organic compounds in feed and permeate solutions were determined by measuring the total organic carbon (TOC) on a TOC analyzer (TOC-V_{CSH}, SHIMADZU).

The rejection rates of each membrane towards the organic solutes with different molecular weights were plotted and fitted by an exponential association equation. The MWCO value was determined at the molecular weight at which the membrane rejection rate equals 90%.

1.8 Calculation of the pore size and pore size distribution of the TFC and TFN membranes

The pore size distribution of the membranes was derived based on the MWCO measurement. The molecular weight of the uncharged organic solutes used in the MWCO measurement was first converted to their Stokes radii. The Stokes radius of DEG (a small neutral organic molecule) r_{DEG} was determined by

$$log r_{DEG} = -1.4962 + 0.4654 log\ (MW_{DEG}) \quad \text{(S7)}$$

where MW_{DEG} is the molecular weight of DEG. The Stokes radii of PEGs (*i.e.*, PEG-200, PEG-400, PEG-600, and PEG-1000) r_{PEG} was calculated by

$$r_{PEG} = 16.73 \times (MW_{PEG})^{0.557} \times 10^{-3} \quad \text{(S8)}$$

where MW_{PEG} is the molecular weight of PEG.

The pore size cutoff curves of the membranes were then plotted based on the rejection performances towards the solutes with different Stokes radii.[1] The pore size distribution of the membranes is assumed to follow the probability density function (PDF) and expressed as

$$\frac{df(r_p)}{d(r_p)} = \frac{1}{r_p ln\sigma_p \sqrt{2\pi}} exp\left[-\frac{(lnr_p - ln\mu_p)^2}{2(ln\sigma_p)^2}\right] \quad \text{(S9)}$$

where r_{p} is the pore size (radius) of the membrane, μ_{p} is the mean effective pore size, and σ_{p} is the geometric standard deviation. Both μ_{p} and σ_{p} were derived from the pore size cutoff

curves of the membranes. The mean pore size is determined at the Stokes radius where 50% of the organic solutes are rejected. The geometric standard deviation follows that of the PDF curve, which is the ratio of the Stokes radius at the 84.13% rejection rate to that at the 50% rejection rate.

2. Characterization results of the CDs

In this work, dodecyltrimethylammonium (C_{12}) and hexadecyltrimethylammonium (C_{16}) ions were also used in the synthesis of C_{12}-CDs and C_{16}-CDs, which flocculate in water because of the hydrophobic long hydrocarbon fragments attached to the CDs surface. The hydrophilicity and lipophilicity of the CDs were further evaluated by dispersing them in DI water and ethanol. Digital images of the dispersions were captured under sunlight and ultraviolet (UV) light (365 nm) and are displayed in Figure S1. C_1-CDs, C_4-CDs, and C_8-CDs are well dispersed in DI water while C_{12}-CDs and C_{16}-CDs are fully dissolved in ethanol to form clear solutions under sunlight and show strong blue emission under UV light.[2] On the other hand, sediment of C_1-CDs and C_4-CDs are observed in their ethanol dispersions while flocculation of C_{12}-CDs and C_{16}-CDs occurs in DI water. The amphiphilic C_8-CDs form a turbid suspension in ethanol without observable sedimentation.

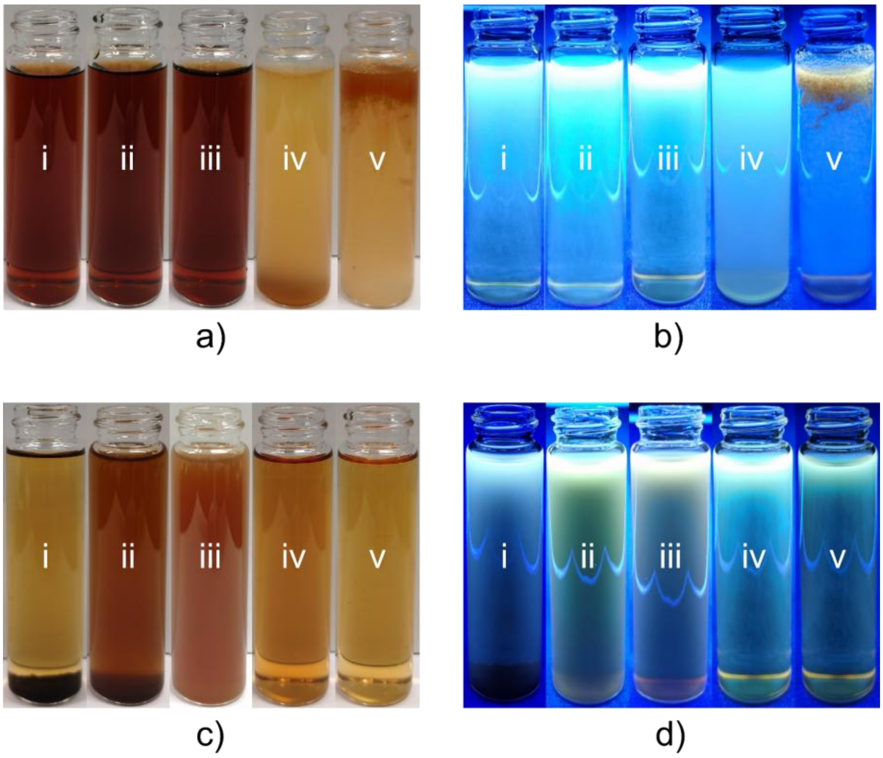

Figure S1. Photos of the ammonium ion–modified CDs dispersed in DI water under a) sunlight and b) UV light (365 nm) and in ethanol under c) sunlight and d) UV light: i) C_1-CDs, ii) C_4-CDs, iii) C_8-CDs, iv) C_{12}-CDs, and v) C_{16}-CDs. All the dispersion were kept under quiescent condition for 2 h for stabilization.

TEM was employed to analyze the size and morphology of the CDs (Figure S2). The size values of the CDs in the TEM images were obtained by ImageJ software. For each size distribution curve, at least 350 nanoparticles were measured for constructing the histogram.

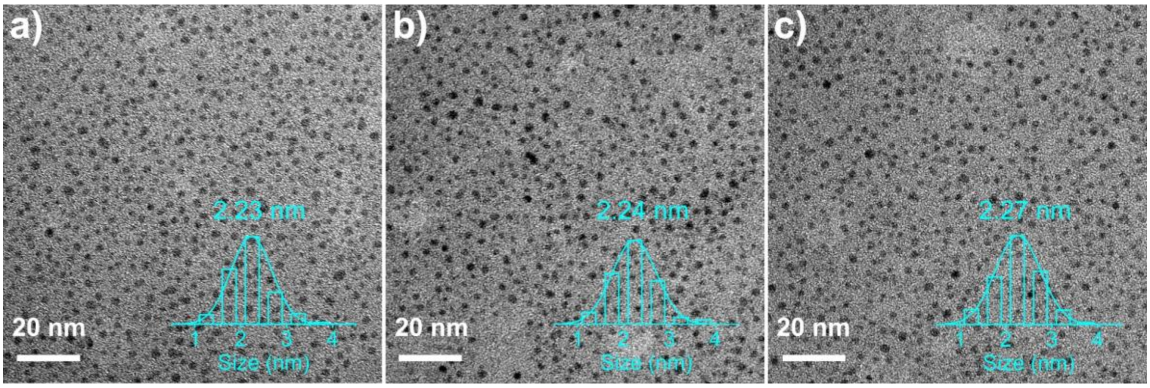

Figure S2. TEM images and size distributions of a) Na-CDs, b) C_1-CDs, and c) C_4-CDs.

The TGA curves were obtained by heating the CDs and ammonium salts used to modify the CDs to 700 °C at a heating rate of 5 °C min^{-1} under Ar gas protection. The data was recorded after the temperature reaches 100 °C to exclude the weight loss due to the evaporation of moisture adsorbed by the super hydrophilic samples.[3] In Figure S3, a drastic drop in the weight ratio of the Na-CDs is observed when the temperature is increased from 250 °C to 500 °C because of the thermal decomposition of the organic functional groups. In contrast, the C_1-CDs, C_4-CDs, and C_8-CDs exhibit deeper declines in the weight ratio when the temperatures are in the ranges of 250–350 °C, 240–300 °C, and 220–260 °C, respectively, as a result of the decomposition of the ammoniums ions attached. The ammonium salts with longer alkyl chains show lower thermostability, and the phenomenon is reflected by the earlier start of thermal decomposition from C_8 to C_1 ions.

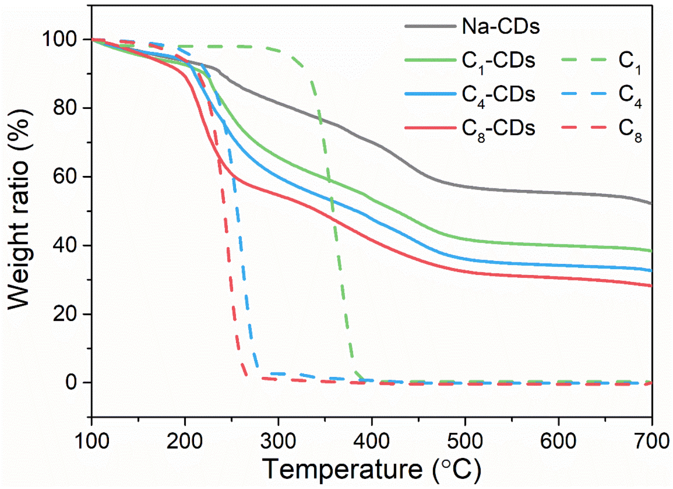

Figure S3. TGA curves of the CDs and ammonium salts.

The XRD pattern of the CDs were obtained by scanning the samples with a 2θ range from 5° to 80° at a step size of 0.02° and a step rate of 10° min^{-1} using Cu-Kα as the radiation source (λ = 1.5418 Å). In Figure S4, the C_1-CDs, C_4-CDs, and C_8-CDs display broad diffraction peaks at 20°, while no distinct peaks are identified in the pattern of Na-CDs probably because of the interference by the large number of Na^+ ions on the CDs surface.

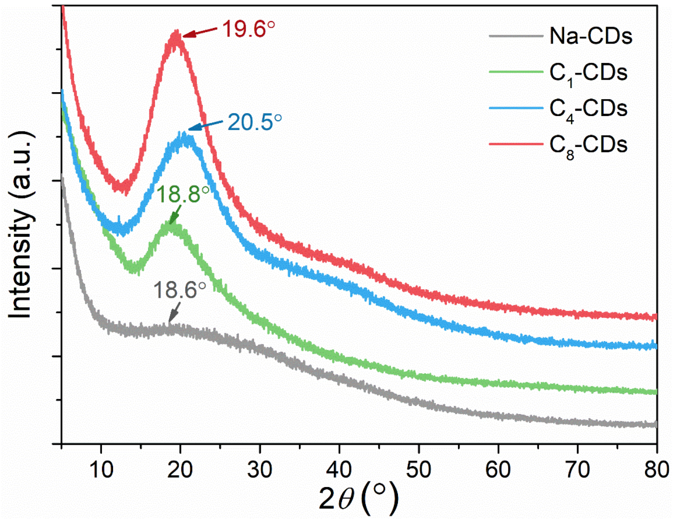

Figure S4. XRD patterns of the CDs.

The XPS analysis was conducted on the four CDs samples to measure their chemical compositions and concentrations. In Table S1, all the CDs show the existence of carbon (C), oxygen (O), and sodium (Na) elements on their surface layers, indicating the successful synthesis of hydrophilic CDs with abundant negatively charged organic functional groups. The appearance of nitrogen (N) in the C_1-CDs, C_4-CDs, and C_8-CDs, together with their much lower concentrations of Na^+ ions than that in the Na-CDs, verifies the attachment of ammoniums ions after the ion-exchange process. The remaining trace amount of Na^+ ions in the ammoniums ion–modified CDs as a result of the incomplete reaction can be attributed to the steric hindrance effect imposed by the ammonium ions, which have much larger hydrated radii than the Na^+ ions.

The high-resolution C 1s and O 1s spectra were deconvoluted to reveal the chemical bonds and their content ratios in the CDs (Figure S5). After the Na^+ ions are substituted by the C_1 ammonium ions, the C_1-CDs display a stronger peak at 286.2 eV, which is contributed by the C–N bond from the tetramethylammonium groups.[4] From C_1-CDs to C_4-CDs and C_8-CDs, the content ratio of the C–C/C=C bond continuously increases while those of the C–O/C–N and C=O bonds continuously decrease as shown in Table S2, because of the longer alkyl chains from C_1 to C_4 and C_8 ammonium ions.

Table S1. Elemental composition and concentration of the surface layer of the CDs.

**CDs**	**Surface elemental composition and concentration**			
	C 1s (%)	N 1s (%)	O 1s (%)	Na 1s (%)
Na-CDs	68.94	–	26.16	4.90
C_1-CDs	75.64	3.04	20.48	0.84
C_4-CDs	77.58	2.50	19.19	0.74
C_8-CDs	79.47	2.42	17.40	0.71

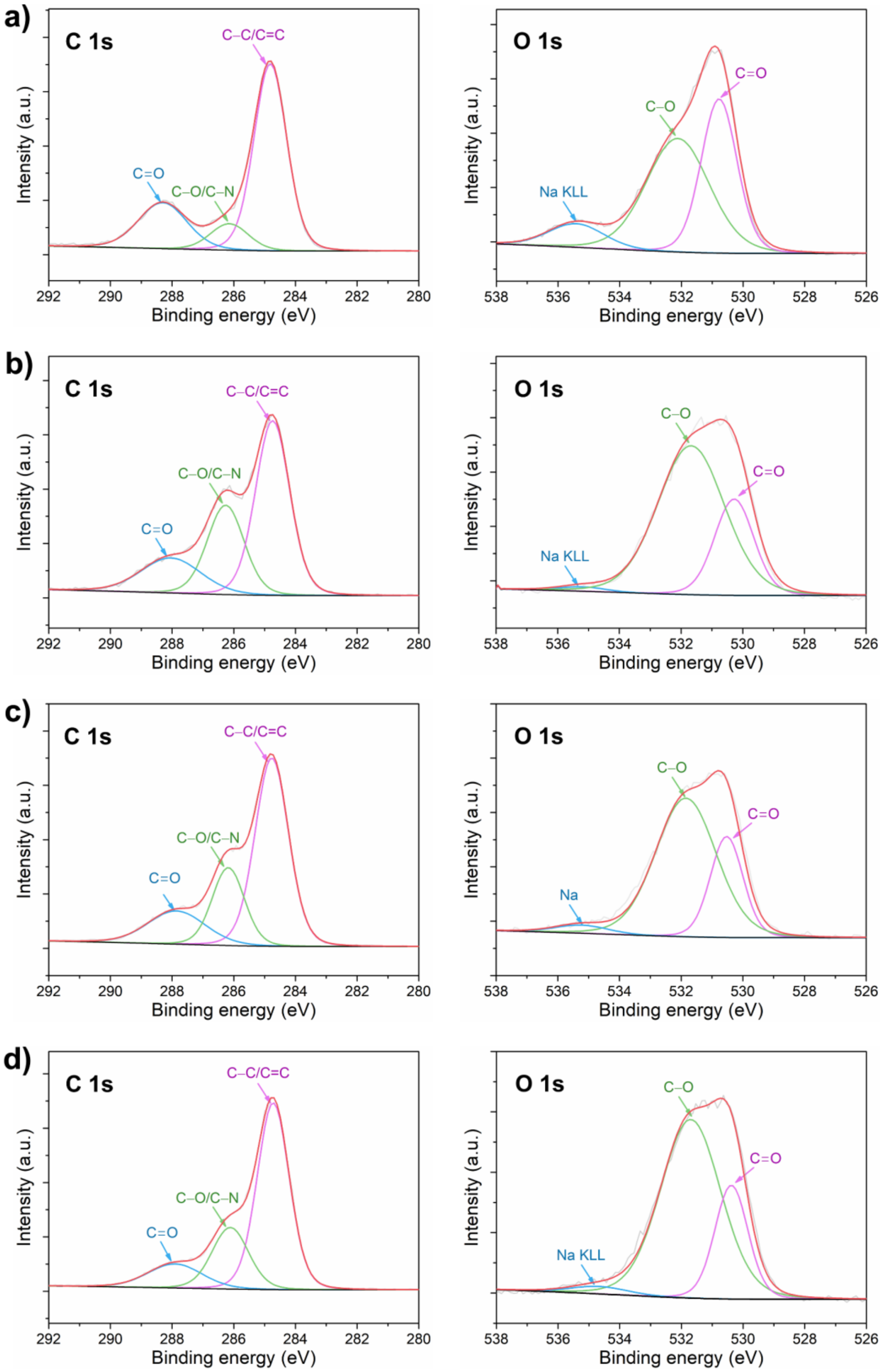

Figure S5. High-resolution C 1s and O 1s XPS spectra of a) Na-CDs, b) C_1-CDs, c) C_4-CDs, and d) C_8-CDs.

Table S2. Chemical bonds and their content ratios in the CDs derived from the high-resolution C 1s and O 1s XPS spectra.

**CDs**	**C 1s (%)**			**O 1s (%)**		
	C–C/C=C 284.8 eV	C–O/C–N 286.2 eV	C=O 288.3 eV	C–O 532.1 eV	C=O 530.8 eV	Na KLL 535.4 eV
Na-CDs	65.46	10.74	23.79	51.23	39.41	9.36
C_1-CDs	52.59	28.94	18.47	71.42	27.10	1.47
C_4-CDs	58.87	23.39	17.74	69.27	27.19	3.54
C_8-CDs	62.81	23.55	13.60	71.93	25.23	2.84

The surface charges of the CDs at pH neutral condition (pH = 7) were obtained by measuring their zeta potentials on a Zetasizer Nano ZS instrument. To ensure the high count rate and good data distribution of the resultant patterns, all the aqueous samples were prepared with a CDs concentration of 0.2 wt %. From Na-CDs to C_1-CDs, C_4-CDs, and C_8-CDs, the zeta potential becomes more negative because of the substitution of the Na^+ ions by the ammonium ions with lower charge density (Figure S6). With the same charge number, the increasing ionic size from Na^+ to C_1, C_4, and C_8 ions leads to the decreasing charge density according to the equation $q = ne/V$, where q is the volume charge density, n is the charge number, e is the charge of the electron (1.60×10^{-19} C), and V is the volume of the ion.

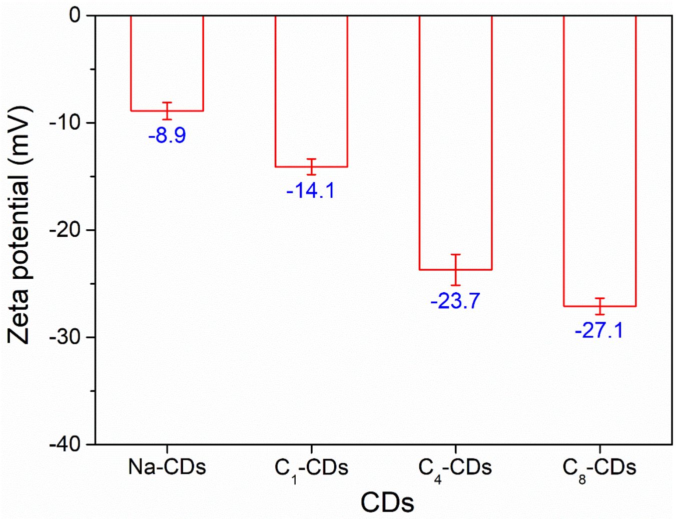

Figure S6. Zeta potentials of the CDs.

3. Characterization results of the membranes

The high-resolution C 1s, N 1s, and O 1s spectra of the TFC and TFN membranes were deconvoluted to reveal the chemical bonds and their content ratios in the membrane surface layers (Figure S7).

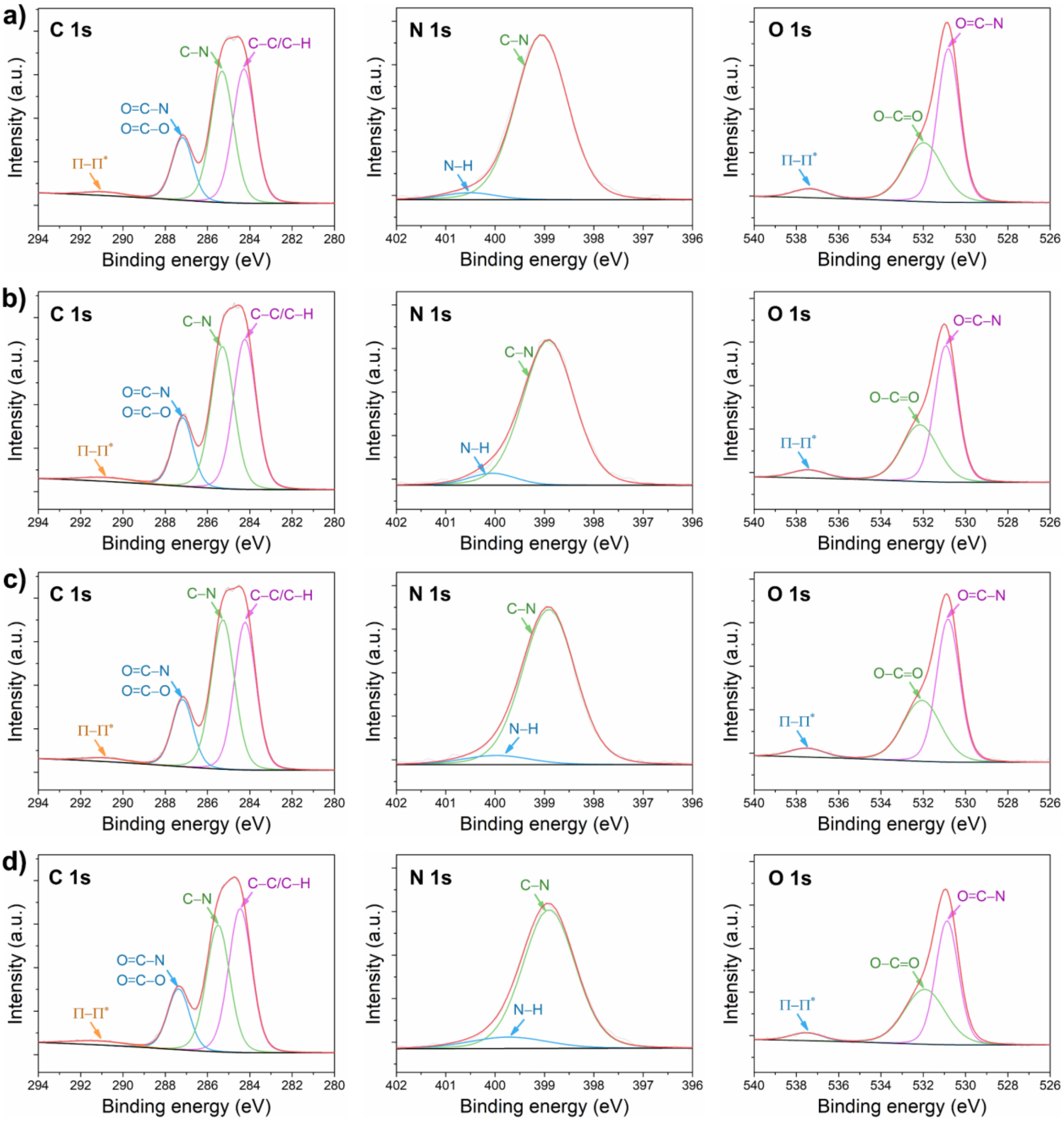

Figure S7. High-resolution C 1s, N 1s, and O 1s XPS spectra of the a) TFC, b) TFN-C_1-CDs, c) TFN-C_4-CDs, and d) TFN-C_8-CDs membranes.

As indicated by the results summarized in Table S3, the content ratios of the N–H bond and O=C–O group generated from the unreacted amine and acyl chloride groups, respectively, increase from the TFC membrane to the TFN-C_1-CDs, TFN-C_4-CDs, and TFN-C_8-CDs membranes. The result indicates that the polyamide layers of the TFN membranes have more unreacted PIP and TMC species than that of the TFC membrane, resulting in the lower degrees of crosslinking for the former polyamide network.[5]

The looser polyamide structure of the TFN membranes is attributed to the decreased kinetics of the IP reaction by the CDs. The negatively charged CDs readily attracted cationic PIP monomer to form the larger CDs/PIP complexes, which rapidly reacted with TMC to generate the polyamide film at the aqueous/organic interface and impede the subsequent diffusion of PIP and TMC monomers towards the reaction interface *via* the steric hinderance effect and electrostatic repulsion.[6] Consequently, the deficit of the reacting monomers led to the incomplete reaction of the amine and acyl chloride groups with their counterpart, and hence the lower degrees of cross-linking.

Table S3. Chemical bonds and their content ratios in the TFC and TFN membranes derived from the high-resolution C 1s, N 1s, and O 1s XPS spectra.

**Membrane**	**C 1s (%)**			**N 1s (%)**		**O 1s (%)**	
	C–C/ C–H	C–N	O=C–O/ O=C–N	C–N	N–H	O=C–O	O=C–N
	284.3 eV	285.3 eV	287.2 eV	399.1 eV	400.5 eV	532.4 eV	530.9 eV
TFC	39.77	42.33	17.90	95.15	4.85	23.46	76.54
TFN-C_1-CDs	43.29	39.63	17.08	92.37	7.64	25.53	74.47
TFN-C_4-CDs	39.37	43.63	16.99	90.79	9.22	26.42	73.58
TFN-C_8-CDs	41.94	40.55	17.51	89.07	10.93	28.06	71.94

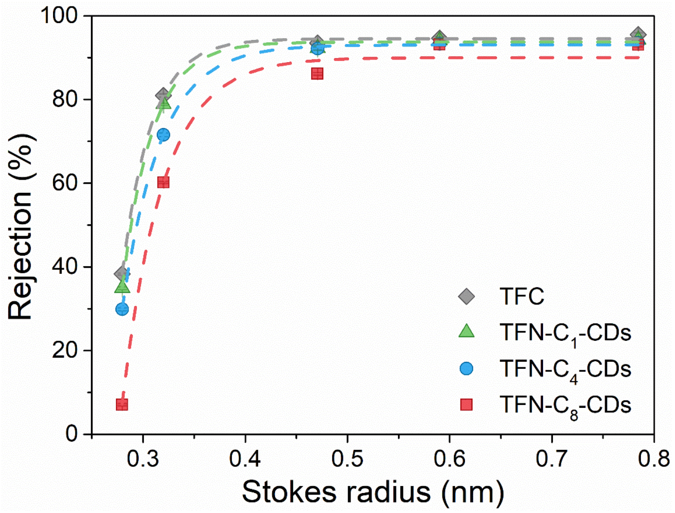

Figure S8. Pore size cutoff curves of the TFC and TFN membranes based on the rejection performance towards the uncharged DEG and PEG molecules.

Table S4. Arithmetic average roughness R_a, height of the nanostructure, and surface area increase of the TFC membrane and the TFN membranes.

**Membrane**	R_a **(nm)**	**Height* (nm)**	**Surface area increase† (%)**
TFC	9.3 ± 0.3	109.3 ± 13.0	11.1 ± 0.1
TFN-C_1-CDs	7.7 ± 0.2	96.3 ± 11.4	9.1 ± 0.2
TFN-C_4-CDs	6.9 ± 0.3	91.3 ± 13.5	7.8 ± 0.1
TFN-C_8-CDs	18.0 ± 0.6	151.2 ± 9.8	24.1 ± 1.1

*The height is calculated as the difference between the highest point and lowest point in the scanned region.
†The surface area increase indicates how much the effective surface area is larger than the scanned area.

To analyze the morphology of the back surface of the TFN-C_8-CDs membrane (*i.e.*, the surface in contact with the PSF substrate), the non-woven fabric support layer was first peeled off from the membrane. The PSF substrate was then dissolved in DMF to form the isolated polyamide layer. In Figure S9, the polyamide layer is highly wrinkled with plenty of trenches, which correspond to the long and narrow ridges on the membrane top surface (Figure 3d). These trenches create voids between the highly dense selective layer to facilitate the fast water transportation by reducing the entry resistance of the water molecules that have been passed through the selective layer into the pores of the PSF substrate underneath. Moreover, the nodular structure of the top surface disappears on the much smoother back surface.

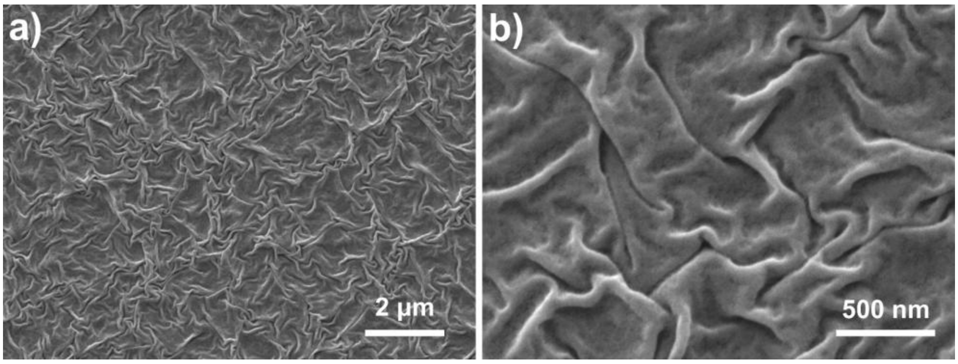

Figure S9. Back surface of the TFN-C_8-CDs membrane: a) the low-magnification and b) the high magnification FE-SEM images.

4. Filtration performances of the membranes

4.1 Effect of the loading concentration of CDs

To investigate the effects of the loading concentrations of the nanoparticles on the filtration performance of the TFN membranes, 0.1–0.6 wt % C_1-CDs, C_1-CDs, and C_1-CDs were dispersed in the aqueous phase for the membrane fabrication. All the filtration tests were conducted using the feed solution of 2000 ppm Na_2SO_4 under 6 bar at room temperature. After the incorporation of CDs modified with ammonium ions into the polyamide layers, the TFN membranes exhibit continuous increases in the water permeability, which can be attributed to the creation of additional water channels by the core–shell structured nanoparticles (Figure S10a). Moreover, at the same loading concentration of CDs, the permeability values exhibit the trend that TFN-C_8-CDs > TFN-C_4-CDs > TFN-C_1-CDs membranes, which is in good accordance with the descending order of the shell size (reflected by the alkyl chain lengths) of the CDs embedded.

On the other hand, defects were produced in the highly wrinkled polyamide layer when an excessive amount of C_8-CDs (*i.e.*, lager than 0.4 wt %) was involved in the membrane preparation, resulting in the sharp drop in the Na_2SO_4 rejection rate of the TFN-C_8-CDs membrane to 95.3% at a loading concentration of 0.5 wt % and 87.1% at 0.6 wt % (Figure S10b). Slight decreases in the salt rejection rates are also observed for the TFN-C_1-CDs and TFN-C_4-CDs membranes with loading concentrations greater than 0.4 wt %. By considering both the permeability and rejection performances, an optimum concentration of 0.4 wt % CDs were used in the preparation of the TFN membranes for all the characterization and filtration tests.

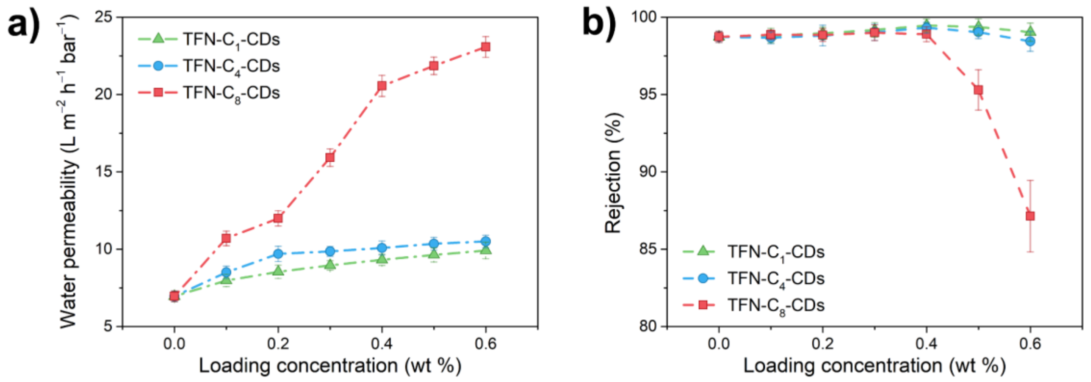

Figure S10. Effects of the loading concentrations of the CDs on the a) water permeability and b) rejection of the TFN membranes.

It was noted that, dissimilar to the logarithmic growth of the water permeability for the TFN-C_1-CDs and TFN-C_4-CDs membranes, an upsurge in the permeability values of the TFN-C_8-CDs membrane occurred when the loading concentration was increased from 0.2 wt % to 0.4 wt %. FE-SEM and AFM analyses were thus conducted to reveal the evolution of the polyamide protrusion on the surface of the TFN-C_8-CDs membrane when the loading concentration was increased from 0.1 wt % to 0.6 wt %. The FE-SEM and AFM images of the TFN-C_8-CDs membranes with different loading concentrations of C_8-CDs are displayed in Figures S11 and S12, respectively. After the introduction of 0.1 wt % C_8-CDs in the IP reaction, the TFN-C_8-CDs-0.1 membrane fabricated has a much smaller R_a value of 5.8 nm (Table S5) than the TFC (11.5 nm), TFN-C_1-CDs (7.7 nm), and TFN-C_4-CDs (6.9 nm) membranes, which suggests that the larger CDs/PIP complexes could effectively slow down the IP reaction to form smoother membrane surfaces (Figures S11a and S12a).

The clusters of polyamide nanoparticles appear and are sparsely distributed on the layer surface when the loading concentration reaches 0.2 wt %, implying that the aggregation of amphiphilic C_8-CDs occurs at higher concentrations (Figures S11b and S12b). The increases in the loading concentration to 0.3 wt % and 0.4 wt % give rise to the formation and growth of the nanosized polyamide strips on the membrane surface (Figures S11c,d and S12c,d),

leading to the significantly larger R_a values of 13.0 nm and 18.0 nm for the TFN-C_8-CDs-0.3 and TFN-C_8-CDs-0.4 membranes, respectively. Meanwhile the surface area increase of the TFN-C_8-CDs-0.4 membrane (24.1%) is more than three times greater than the TFN-C_8-CDs-0.1 membrane (7.5%). The ridges with leaf-like structure are obtained after the further addition of C_8-CDs (*i.e.*, loading concentrations of 0.5 wt % and 0.6 wt %), bringing the further increased R_a values of 26.2 nm and 24.9 nm, while the valley regions become smoother as a result of the local deficit of PIP monomers (Figures S11e,f and S12e,f).

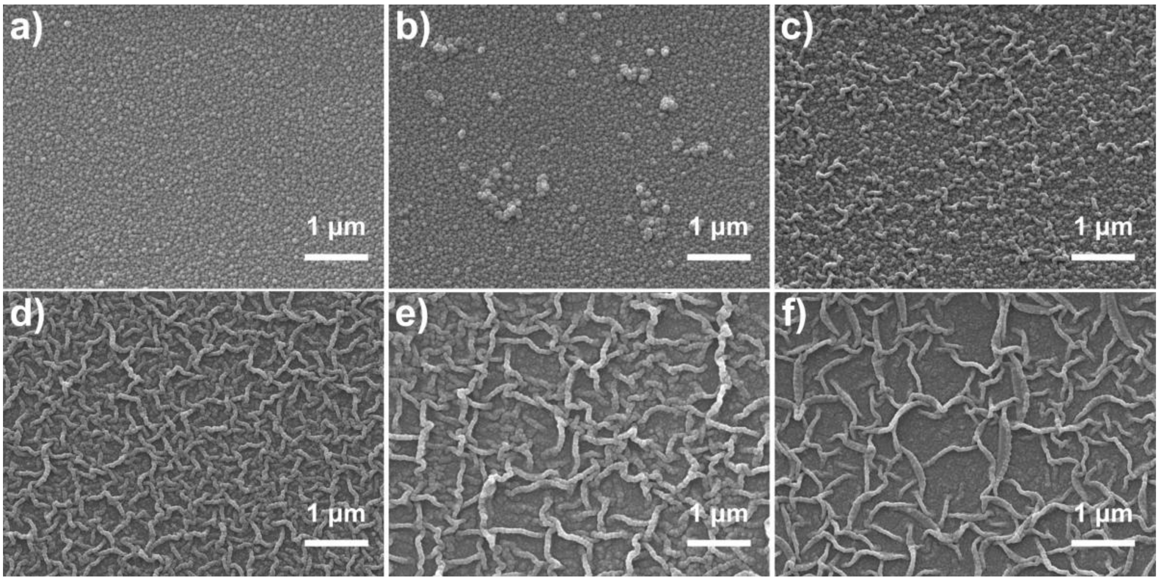

Figure S11. FE-SEM images of the surface morphology of the TFN-C_8-CDs membranes with different loading concentrations of C_8-CDs: a) 0.1 wt %, b) 0.2 wt %, c) 0.3 wt %, d) 0.4 wt %, e) 0.5 wt %, and f) 0.6 wt %.

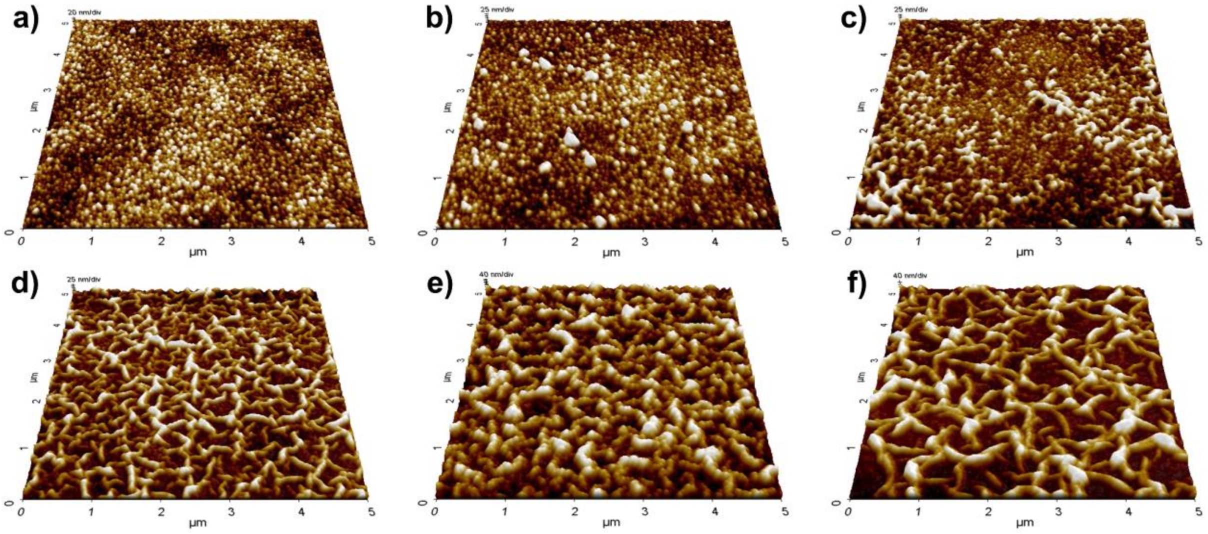

Figure S12. AFM images of the TFN-C_8-CDs membranes with different loading concentrations of C_8-CDs: a) 0.1 wt %, b) 0.2 wt %, c) 0.3 wt %, d) 0.4 wt %, e) 0.5 wt %, and f) 0.6 wt %.

Table S5. Arithmetic average roughness R_a, height of the nanostructure, and surface area increase of the TFN-C_8-CDs membranes with different loading concentrations of C_8-CDs.

**Membrane**	**Loading concentration of C_8-CDs (wt %)**	**R_a (nm)**	**Height (nm)**	**Surface area increase (%)**
TFN-C_8-CDs-0.1	0.1	5.8 ± 0.2	75.4 ± 14.3	7.5 ± 1.1
TFN-C_8-CDs-0.2	0.2	7.9 ± 0.2	90.8 ± 5.5	9.0 ± 1.2
TFN-C_8-CDs-0.3	0.3	13.0 ± 0.2	132.7 ± 4.6	20.3 ± 2.7
TFN-C_8-CDs-0.4	0.4	18.0 ± 0.6	151.2 ± 9.8	24.1 ± 1.1
TFN-C_8-CDs-0.5	0.5	26.2 ± 0.7	210.7 ± 26.7	33.2 ± 2.9
TFN-C_8-CDs-0.6	0.6	24.9 ± 0.8	206.2 ± 31.4	26.2 ± 2.6

The effect of C_8-CDs concentration on the NaCl/Na_2SO_4 ion selectivity $S_{\mathrm{NaCl/Na_2SO_4}}$ and pure water permeability (different from the water permeability when treating salt water) of the TFN-C_8-CDs membranes was investigated using 2000 ppm NaCl, 2000 ppm Na_2SO_4, or pure water as the feed solutions under a constant pressure of 6 bar at room temperature. As the loading concentration of the nanoparticles increases from 0 to 0.4 wt %, the rejection to Na_2SO_4 is well maintained while the NaCl rejection drastically decreases from 28% to 10.6% (Figure S13a). The NaCl rejection further decreases to 5.2% when the loading concentration reaches 0.6 wt %. Meanwhile, the pure water permeability continuously increases from 9.1 L m^{-2} h^{-1} bar^{-1} to 37.7 L m^{-2} h^{-1} bar^{-1}, accompanied with an initially improved selectivity from 56.7 (0 wt %) to 87.3 (0.3 wt %) and a drastic decrease from 83.5 (0.4 wt %) to 7.4 (0.6 wt %), as shown in Figure S13b.

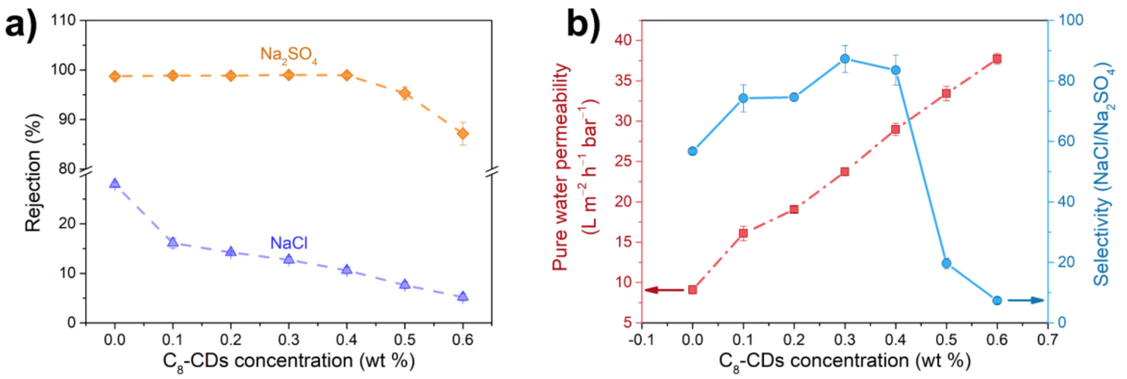

Figure S13. Effects of the loading concentration of C_8-CDs on the a) salt rejection and b) pure water permeability and NaCl/Na_2SO_4 ion selectivity of the TFN-C_8-CDs membrane.

4.2 Effect of the concentrations of PIP and TMC

To investigate the effects of the PIP and TMC concentrations on the filtration performance and surface morphology (*i.e.*, the ridge-and-valley structure) of the TFN-C_8-CDs membrane, 0.5–1.5 wt % PIP (the concentration of TMC was fixed at 0.15 wt %) and 0.05–0.25 wt % TMC (the concentration of PIP was fixed at 1.0 wt %) were used for the IP reaction. The filtration tests were conducted using 2000 ppm Na_2SO_4, 2000 ppm NaCl, or pure water as the feed solution under 6 bar at room temperature. The surface morphology of the fabricated membranes was obtained by FE-SEM.

As shown in Figure S14a,b, the increase in the PIP concentration from 0.5 wt % to 1.5 wt % leads to the continuous increase in both the Na_2SO_4 and NaCl rejection, resulting in the escalated NaCl/Na_2SO_4 ion selectivity from 6.2 to 136.3, while the pure water permeability declines from 48.7 L m^{-2} h^{-1} bar^{-1} to 21.3 L m^{-2} h^{-1} bar^{-1}. The primary cause of the trade-off relation between the pure water permeability and NaCl/Na_2SO_4 selectivity is the variation of the structure density of the selectively layer. A low PIP concentration brings a loose polyamide structure that allows fast transportation of both water molecules and salt ions. Moreover, the change in the surface morphology could affect the permeability and rejection performances of the TFN-C_8-CDs membranes (Figure S15).

At a low PIP concentration of 0.5 wt %, thin nanosized strips are formed on a relatively smooth polyamide layer, indicating a mild IP reaction process. As the concentration increases, more PIP monomers are attracted to the negatively charged C_8-CDs to form larger accumulation of C_8-CDs/PIP complexes. Consequently, the strips become thicker, while new polyamide nanoparticles evolve and form aggregates and strands in the smooth valley regions as PIP is no longer in deficit in these regions. Although the surface roughness and hence the area increase of the membrane fabricated with a lower PIP concentration is smaller than that fabricated with a higher concentration, the smaller membrane thickness imposes smaller

energy burden for the passage of water molecules and salt ions. Meanwhile, because of the presence of large number of C_8-CDs in the selectively layer, the thin polyamide layer, especially the ridges where C_8-CDs aggregate, is prone to the formation of unselective defects, resulting in the low ion-sieving capability and NaCl/Na_2SO_4 selectivity.

Figure S14c,d shows that, contrary to the effect of the PIP concentration, the increase of the TMC concentration from 0.05 wt % to 0.25 wt % deteriorates the ion rejection rates, hence the decrease in the NaCl/Na_2SO_4 selectivity from 110.0 to 17.4, and enhances the pure water permeability from 12.8 L m^{-2} h^{-1} bar^{-1} to 32.2 L m^{-2} h^{-1} bar^{-1}. The reverse trend suggests that not only a high PIP concentration but also a high concentration ratio of PIP to TMC are essential to the formation of a dense polyamide layer embedded with amphiphilic C_8-CDs, because the polyamide chains formed by the reaction between PIP and TMC with a stoichiometric ratio of 1 : 1 require an additional PIP molecule to crosslink each pair of the repeating units from two adjacent chains. As the TMC concentration increases, a larger number of acyl groups remain unreacted in the polyamide chains, which bring a looser polyamide structure to facilitate the passage of both solvents and solutes.

The FE-SEM images of the membranes fabricated using different TMC concentrations are displayed in Figure S16. The membrane prepared with a low TMC concentration of 0.05 wt % is even smoother than that fabricated using a low PIP concentration of 0.5 wt % with thicker polyamide strips. At higher TMC concentrations, the strips become rougher with their thickness remain largely unchanged, while an increasing number of polyamide nodules (the secondary or grafted nodules) occupy the valley regions. The larger membrane filtration area as indicated by the larger surface roughness, together with the higher chance of producing defects on the coarser polyamide surface, contributes to the higher water permeability and lower salt rejection of the TFN-C_8-CDs membrane.

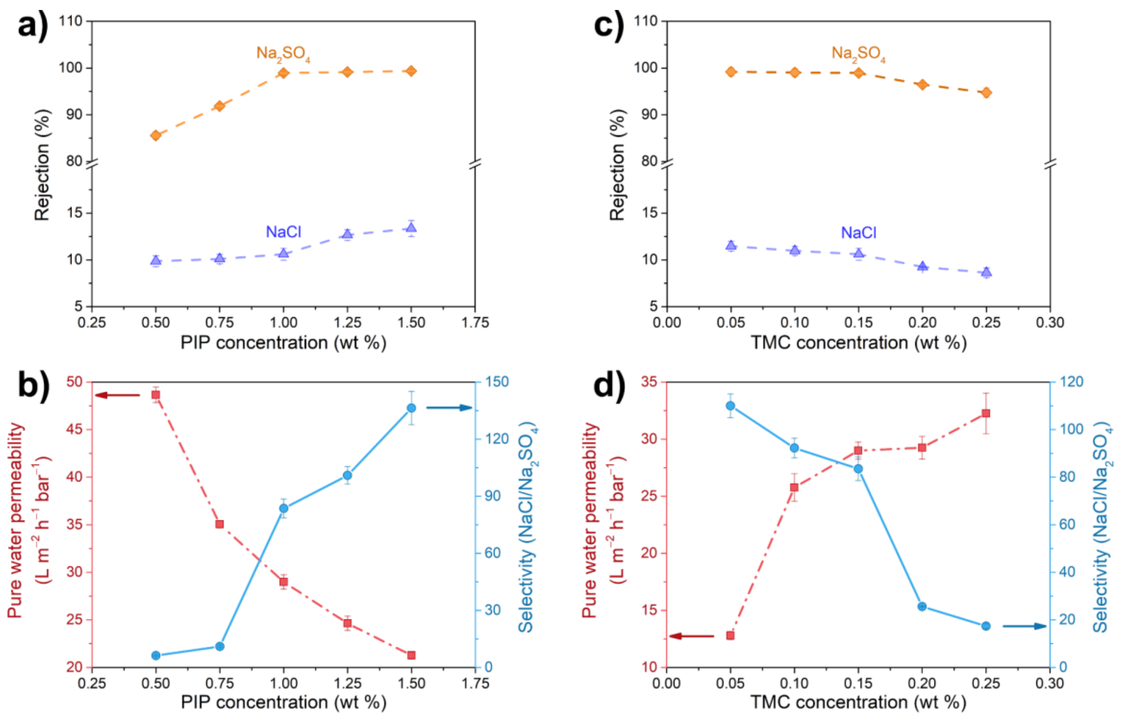

Figure S14. Effects of the concentrations of a,b) PIP and c,d) TMC on the salt rejection, pure water permeability, and NaCl/Na_2SO_4 ion selectivity of the TFN-C_8-CDs membrane. The TMC concentration was fixed at 0.15 wt % in a,b) while the PIP concentration was fixed at 1.0 wt % in c,d).

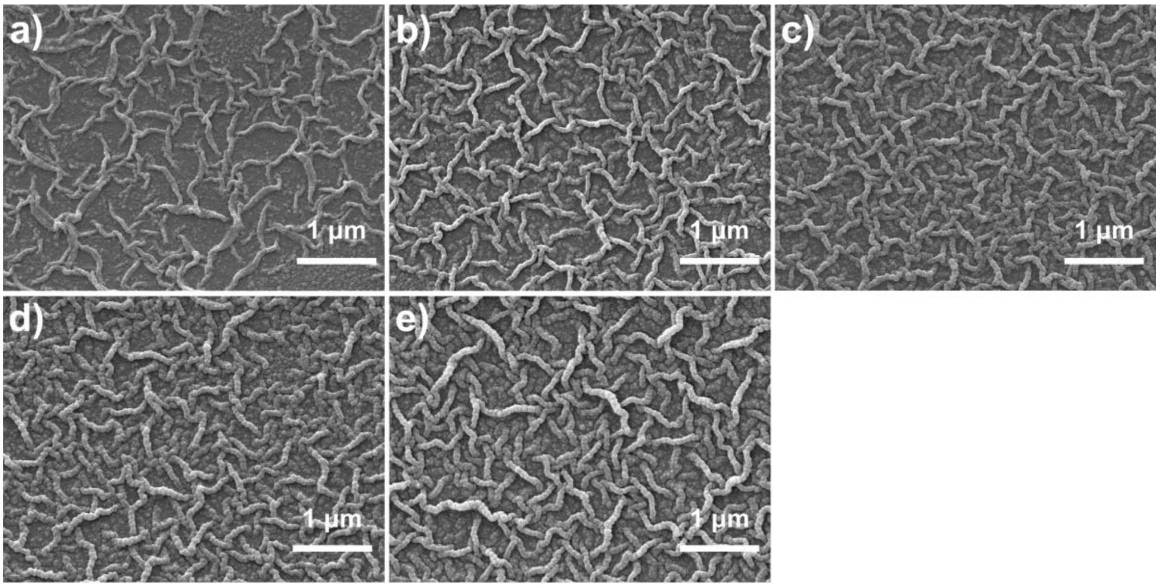

Figure S15. FE-SEM images of the surface morphology of the TFN-C_8-CDs membranes prepared using different PIP concentrations: a) 0.5 wt %, b) 0.75 wt %, c) 1.0 wt %, d) 1.25 wt %, and e) 1.5 wt %. The TMC concentration was fixed at 0.15 wt %.

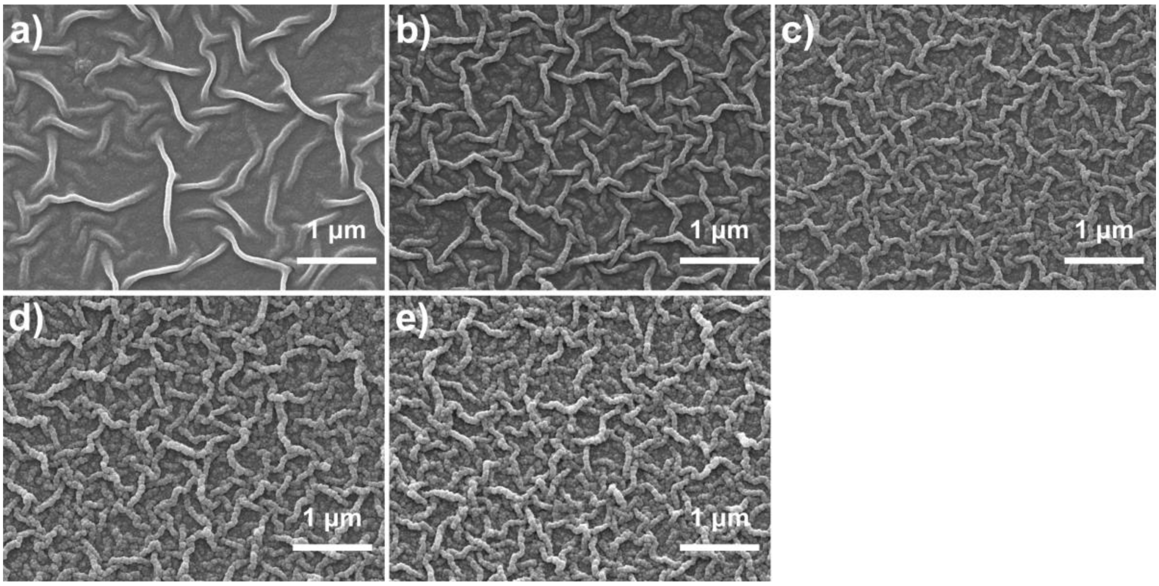

Figure S16. FE-SEM images of the surface morphology of the TFN-C_8-CDs membranes prepared using different TMC concentrations: a) 0.05 wt %, b) 0.1 wt %, c) 0.15 wt %, d) 0.2 wt %, and e) 0.25 wt %. The PIP concentration was fixed at 1.0 wt %.

4.3 Effect of the concentration ratio of C_8-CDs to C_8

The mechanisms of the enhanced pure water permeability and the NaCl/Na_2SO_4 ion selectivity of the TFN-C_8-CDs membrane were studied by varying the concentration ratio of C_8-CDs and C_8. The concentrations of PIP and TMC were fixed at 1.0 wt % and 0.15 wt %, respectively. All the filtration tests were conducted using 2000 ppm Na_2SO_4, 2000 ppm NaCl, or pure water as the feed solution under 6 bar at room temperature. The surface morphology of the fabricated membranes was revealed by FE-SEM.

With a fixed total concentration of 0.4 wt %, the increase in the concentration of C_8-CDs from 0 to 0.4 wt % effectively drives up the membrane permeability from 12.4 L m^{-2} h^{-1} bar^{-1} to 29.0 L m^{-2} h^{-1} bar^{-1} with the selectivity increased from 57.7 to 83.5, which is attributed to the maintained Na_2SO_4 rejection and declined NaCl rejection (Figure S17a,b). The results suggest that the enhanced permeability performance of the TFN-C_8-CDs membrane is mainly attributed to the embedment of the core–shell structured C_8-CDs. It is noted that the involvement of C_8 ammonium ions in the IP reaction slightly improves the permeability performance but lowers both the Na_2SO_4 and NaCl rejection and hence the NaCl/Na_2SO_4 selectivity of the membranes with the same loading concentration of C_8-CDs by comparing the results with those in Figure S13.

To further confirm the deterioration of the membrane selectivity by the cationic ammonium ions, 0.1–0.4 wt % C_8 were added into the aqueous phase of 1.0 wt % PIP and 0.4 wt % C_8-CDs. A higher concentration of Na_2SO_4 and NaCl passes through the membrane while the permeability is slightly enhanced when the dosage of C_8 increases from 0 wt % to 0.4 wt % (Figure S17c,d). The decline in the membrane selectivity can be explained by the impedance imposed by the cationic ammonium ions on the similarly charged PIP monomers during the IP reaction.[5] Compared with PIP, the amphiphilic C_8 ammonium ion with a hydrophilic ammonium head and a hydrophobic alkyl chain are a good emulsifier of water

and n-hexane. When the aqueous solution in the surface layer of substrate is brought in contact with organic phase, the C_8 ions can readily penetrate the water/n-hexane interface while hindering the diffusion of PIP *via* electrostatic repulsion, leading to a slower and more heterogenous IP reaction.[7] As a consequence, more free volumes are created in the polyamide layer and the negative membrane surface charge could be reduced by the ammonium ions, whereby the energy barrier for the diffusion of salt ions through the membrane is reduced.

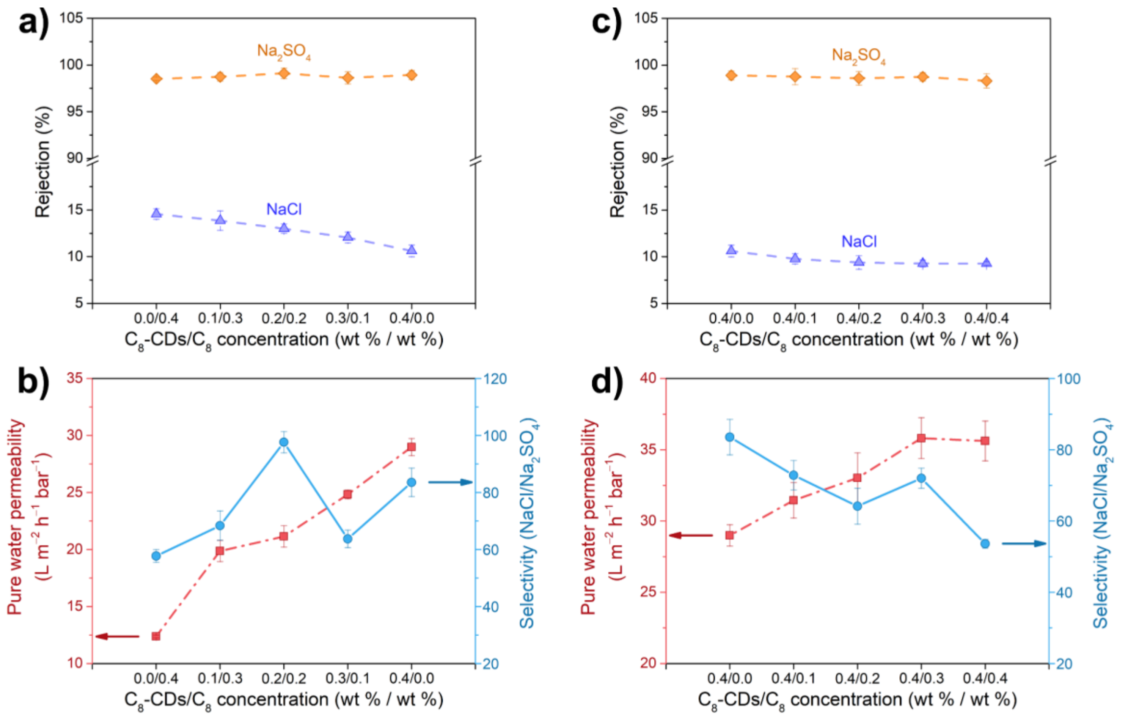

Figure S17. Effect of the concentration ratio of C_8-CDs to C_8 on the salt rejection, pure water permeability, and NaCl/Na_2SO_4 ion selectivity of the TFN-C_8-CDs membrane: a) the total concentration of C_8-CDs and C_8 was fixed at 0.4 wt % and b) the concentration of C_8-CDs was fixed at 0.4 wt %.

FE-SEM was conducted to better understand the effect of the C_8 ammonium ions on the surface morphology of the TFN-C_8-CDs membrane (Figure S18). With a fixed total loading concentration of 0.4 wt %, smooth membrane surfaces are obtained when the concentration of C_8-CDs is below 0.2 wt %, indicating that the C_8 ammonium ions have negligible

contribution to the formation of crumpled polyamide layers. As the concentration of C_8-CDs goes beyond 0.2 wt %, the aggregates and strands of the polyamide nanoparticles emerge on the membrane surface and grow into smoother polyamide strips. When the concentration of C_8-CDs is fixed at 0.4 wt %, the addition of C_8 ammonium ions from 0.1 wt % to 0.4 wt % results in thicker polyamide ridges on the membrane surface with larger and smoother valley regions. The cationic C_8 ammonium ions in the aqueous phase effectively repel the PIP monomers, causing the higher concentration of PIP in the PIP/C_8-CDs complexes (correspond to the thicker ridges) and the deficit of PIP in other regions (correspond to the smoother valley regions).

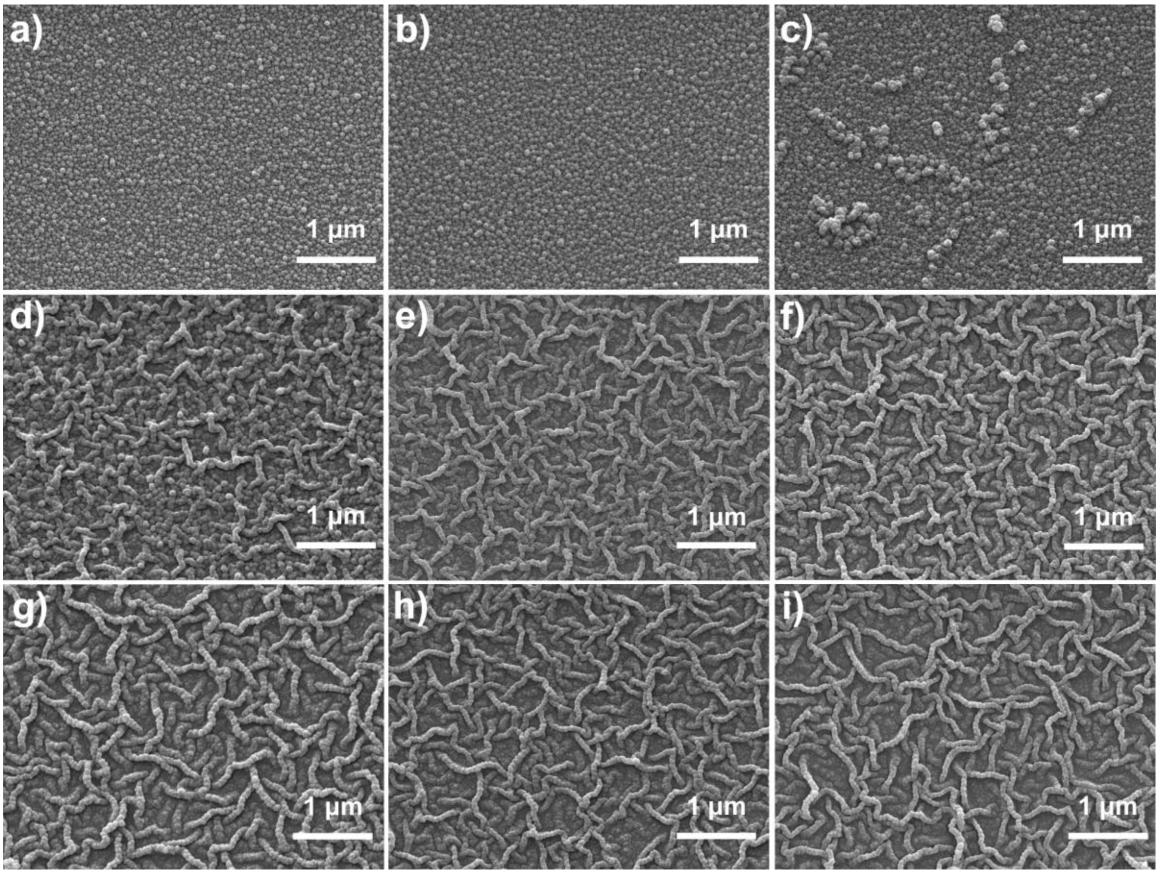

Figure S18. FE-SEM images of the surface morphology of the TFN-C_8-CDs membranes prepared using different concentration ratios of C_8-CDs to C_8 (C_8-CDs/C_8): a) 0.0/0.4, b) 0.1/0.3, c) 0.2/0.2, d) 0.3/0.1, e) 0.4/0.0, f) 0.4/0.1, g) 0.4/0.2, h), 0.4/0.3, and i) 0.4/0.4 wt %/wt %.

4.4 Filtration stability of the TFN membranes

To evaluate the stability of the filtration performances, the permeate flux and rejection rates of the TFC and TFN membranes towards Na_2SO_4 solutions were measured by varying the applied pressure, salt concentration, and operating temperature. The flux values of all the membranes show high linearity under a wide range of pressure from 2 bar to 10 bar, while the rate of the flux increase with the applied pressure follow the trend: TFN-C_8-CDs > TFN-C_4-CDs > TFN-C_1-CDs > TFC (Figure S19a). The more rapid increases of the flux for the TFN membranes than that for the TFC membrane implies that the lower entry resistance of the nano-sized channels created by the core–shell structured CDs facilitates the faster transportation of water molecules through the dense polyamide layer under a larger pressure difference across the membrane. Meanwhile, the rejection performance is improved when the pressure is increased, because the relatively constant salt flux through the membrane is diluted by the enhanced water flux (Figure S19b).

A higher salt concentration leads to the slightly decreased permeate flux of all the four membranes, which could be explained by the intensified concentration polarization effect at the feed side (Figure S19c). Under the same applied pressure, the incremented osmotic pressure induced across the semi-permeable membrane by the more concentrated salt solution at the feed side raises the energy barrier to the water flux.[8] The lower rejection of all the membranes under a higher Na_2SO_4 concentration is due to the charge screening effect (Figure S19d). As the salt concentration increases from 500 ppm to 2500 ppm, more Na^+ ions are attracted and adsorbed by the negatively charged membrane surface and nanochannels to reduce the membrane charge density. Moreover, the Donnan effect, a major contributor to the membrane selectivity, significantly decreases with an increasing electrolyte concentration.[9] Consequently, the lower energy barrier due to the weakened electrostatic repulsion facilitates the transportation of the SO_4^{2-} ions through the membranes.

The increase in the operating temperature from 25 °C to 60 °C boosts the permeate flux while slightly reducing the rejection rates of the membranes (Figure S19e,f). The highest and the lowest rates of the increase in flux is observed for the TFN-C_8-CDs and TFC membrane, respectively, while the TFN-C_4-CDs membrane shows a slightly higher rate of increase than the TFN-C_1-CDs membrane. It is postulated that the more rapid flux increases of the TFN membranes than that of the TFC membrane are mainly due to the faster water transportation within the expanded nanochannels as a result of the intensified vibration of the ammonium ions.[10] Moreover, the water solution has a lower viscosity at an elevated temperature, which allows the accelerated transportation of water molecules through the polyamide layer.[11] Meanwhile, the salt rejection of the TFN membranes declines more significantly than that of the TFC membrane, indicating a partial loss of the sieving capacity of the selective layers due to the pore expansion. Nonetheless, the TFN-C_8-CDs membrane still maintains a high rejection rate of 97.3% against 2000 ppm Na_2SO_4 solution at a high temperature of 60 °C.

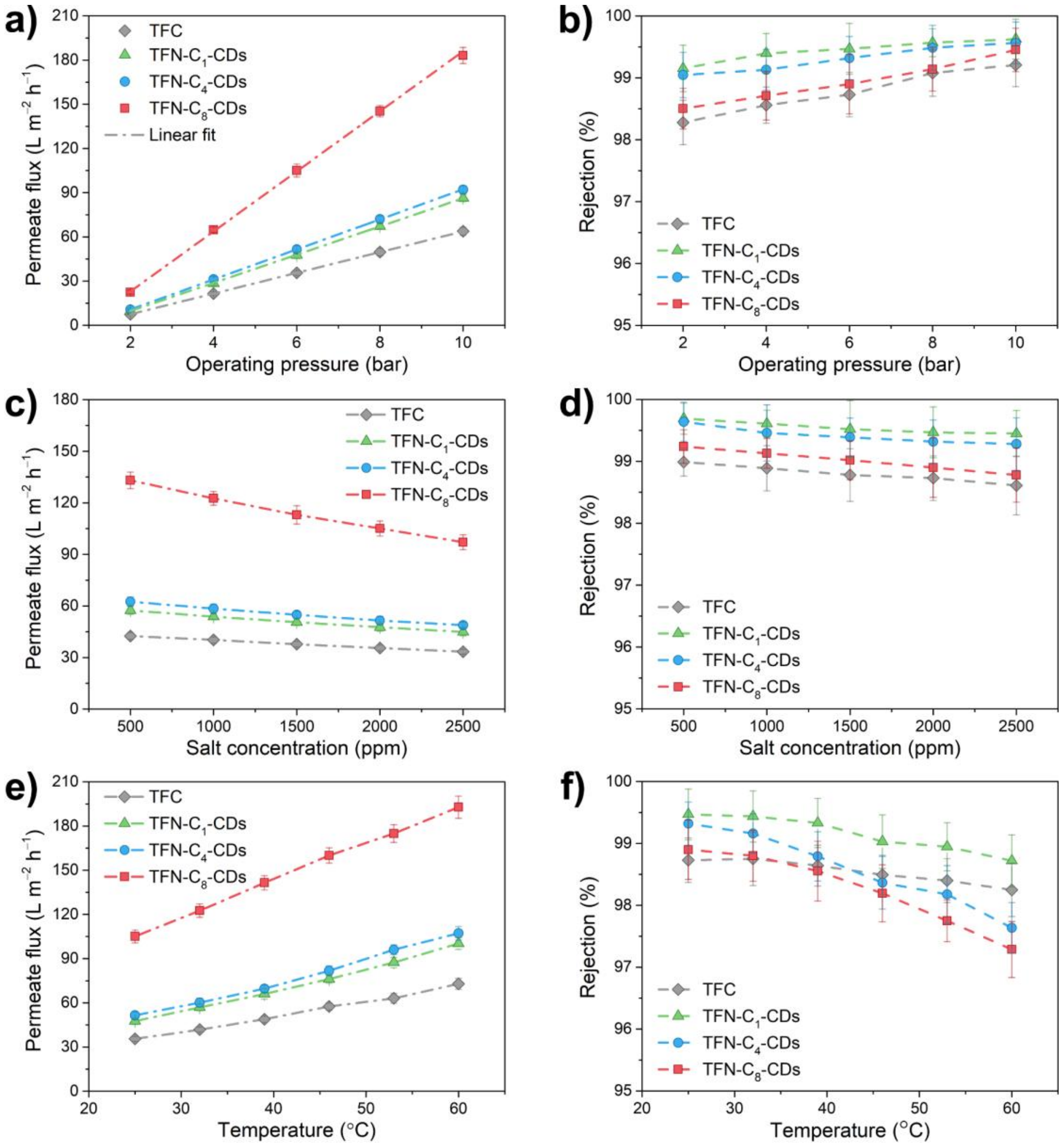

Figure S19. Filtration performance of the TFC and TFN membranes under different operating conditions: a, b) applied pressure; c, d) salt concentration; d, e) operating temperature.

The stability of the pore structure and surface morphology of the TFN membranes for the rapid water transportation under various filtration conditions was further assessed by normalizing the permeate flux of the TFN membranes by that of the TFC membrane. As displayed in Figure S20, the permeate flux ratios of all the TFN membranes are highly stable under a wide range of applied pressures, salt concentrations, and operating temperatures, indicating that the core–shell structured CDs and the surrounding polyamide structure remain intact, and the water channels and surface nanostructure created have negligible deformation under a harsh operating environment. In particular, the TFN-C_8-CDs membrane with the largest pore size and effective filtration area demonstrates roughly two-times higher permeate flux than the TFC membrane, while the TFN-C_1-CDs and TFN-C_4-CDs membranes show around 34% and 45% increases in the flux, respectively.

However, when treating solutions of different salts and organic solutes, the TFN membranes exhibit fluctuations in the permeate flux ratios. Specifically, the ratios of the TFN membranes for the treatment of $MgSO_4$, $MgCl_2$, MB, and CV are lower than those for the treatment of the Na salts and anionic dye molecules. The relatively lower ratios could be ascribed to the faster transportation of the divalent Mg^{2+} ions and cationic dye molecules towards the negatively charged membrane surfaces and through the water channels created by anionic CDs. The competition between the diffusion of solutes and water molecules through the polyamide selective layer results in a smaller water flux. Moreover, the large Mg^{2+} (a Stokes radius of 0.347 nm)[5] and dye ions (a Stoke radius of 0.486 nm for MB[12] and 0.655 nm for CV[13]) may block the pores in the TFN membranes and impedes the water transportation. Nonetheless, the TFN membrane still exhibits higher permeate flux values than the TFC membranes, with the flux ratios ranging from 1.18 to 1.35 for the TFN-C_1-CDs membrane, 1.31 to 1.50 for the TFN-C_4-CDs membrane, and 2.53 to 3.10 for the TFN-C_8-CDs membrane.

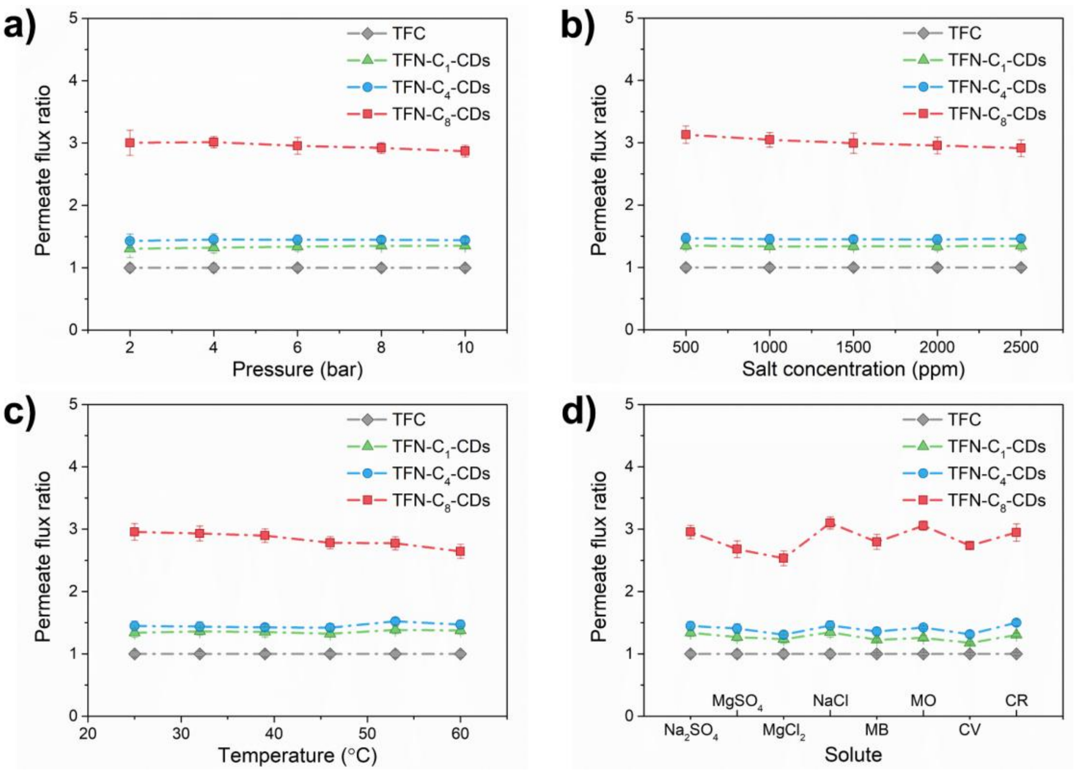

Figure S20. Permeate flux ratios of the TFN membranes to the TFC membrane under various filtration conditions: a) the applied pressure, b) the salt concentration, c) the operating temperature, and d) the type of solutes.

REFERENCES

[1] X. Cheng, Y. Qin, Y. Ye, X. Chen, K. Wang, Y. Zhang, A. Figoli, E. Drioli, *Chem. Eng. J.* **2021**, *417*, 127976.
[2] H. Yang, Y. Liu, Z. Guo, B. Lei, J. Zhuang, X. Zhang, Z. Liu, C. Hu, *Nat. Commun.* **2019**, *10*, 1789.
[3] H. Zheng, Z. Mou, K. Zhou, *ACS Appl. Mater. Interfaces* **2020**, *12*, 53215.
[4] Q. Shen, Y. Lin, Y. Kawabata, Y. Jia, P. Zhang, N. Akther, K. Guan, T. Yoshioka, H. Shon, H. Matsuyama, *ACS Appl. Mater. Interfaces* **2020**, *12*, 38662.
[5] Y. Liang, Y. Zhu, C. Liu, K. R. Lee, W. S. Hung, Z. Wang, Y. Li, M. Elimelech, J. Jin, S. Lin, *Nat. Commun.* **2020**, *11*, 2015.
[6] S. Han, Z. Wang, S. Cong, J. Zhu, X. Zhang, Y. Zhang, *J. Mater. Chem. A* **2020**, *8*, 25028.
[7] C. Jiang, L. Tian, Z. Zhai, Y. Shen, W. Dong, M. He, Y. Hou, Q. J. Niu, *J. Membr. Sci.* **2019**, *589*, 117244.
[8] H. Peng, W. H. Zhang, W. S. Hung, N. Wang, J. Sun, K. R. Lee, Q. F. An, C. M. Liu, Q. Zhao, *Adv. Mater.* **2020**, *32*, 2001383.
[9] Y. L. Ji, B. X. Gu, S. J. Xie, M. J. Yin, W. J. Qian, Q. Zhao, W. S. Hung, K. R. Lee, Y. Zhou, Q. F. An, *Adv. Mater.* **2021**, *33*, 2102292.
[10] J. R. Werber, C. O. Osuji, M. Elimelech, *Nat. Rev. Mater.* **2016**, *1*, 16018.
[11] K. Tiwari, P. Sarkar, S. Modak, H. Singh, S. K. Pramanik, S. Karan, A. Das, *Adv. Mater.* **2020**, *32*, 1905621.
[12] S. Cheng, D. L. Oatley, P. M. Williams, C. J. Wright, *Water Res.* **2012**, *46*, 33.
[13] J. Pang, X. Cui, Y. Feng, Z. Guo, G. Kong, L. Yu, C. Zhang, R. Wang, Z. Kang, D. Sun, *Sep. Purif. Technol.* **2022**, *278*, 119504.
[14] Y. Zeng, L. Wang, L. Zhang, J. Q. Yu, *J. Membr. Sci.* **2018**, *546*, 225.
[15] Y.-L. Ji, Q.-F. An, Y.-S. Guo, W.-S. Hung, K.-R. Lee, C.-J. Gao, *J. Mater. Chem. A* **2016**, *4*, 4224.
[16] N. Dizge, R. Epsztein, W. Cheng, C. J. Porter, M. Elimelech, *J. Membr. Sci.* **2018**, *549*, 357.
[17] Y.-J. Tang, Z.-L. Xu, S.-M. Xue, Y.-M. Wei, H. Yang, *J. Membr. Sci.* **2016**, *498*, 374.
[18] J. Wang, Y. Wang, J. Zhu, Y. Zhang, J. Liu, B. Van der Bruggen, *J. Membr. Sci.* **2017**, *533*, 279.
[19] X. Yang, *ACS Omega* **2019**, *4*, 13824.
[20] C. Wang, Z. Li, J. Chen, Z. Li, Y. Yin, L. Cao, Y. Zhong, H. Wu, *J. Membr. Sci.* **2017**, *523*, 273.
[21] R. Dai, X. Wang, C. Y. Tang, Z. Wang, *Environ. Sci. Technol.* **2020**, *54*, 7619.
[22] M. Zhang, J. Sun, Y. Mao, G. Liu, W. Jin, *J. Membr. Sci.* **2019**, *574*, 196.
[23] J. Zhu, L. Qin, A. Uliana, J. Hou, J. Wang, Y. Zhang, X. Li, S. Yuan, J. Li, M. Tian, *ACS Appl. Mater. Interfaces* **2017**, *9*, 1975.
[24] Y. Pan, R. Xu, Z. Lü, S. Yu, M. Liu, C. Gao, *J. Membr. Sci.* **2017**, *523*, 282.
[25] Z. Zhang, G. Kang, H. Yu, Y. Jin, Y. Cao, *J. Membr. Sci.* **2019**, *570*, 403.
[26] Z. Zhai, C. Jiang, N. Zhao, W. Dong, H. Lan, M. Wang, Q. J. Niu, *J. Mater. Chem. A* **2018**, *6*, 21207.

[27] M. Wu, T. Ma, Y. Su, H. Wu, X. You, Z. Jiang, R. Kasher, *J. Membr. Sci.* **2017**, *544*, 79.

[28] Y. Li, E. Wong, Z. Mai, B. Van der Bruggen, *J. Membr. Sci.* **2019**, *592*, 117396.

[29] H. Sun, J. Liu, X. Luo, Y. Chen, C. Jiang, Z. Zhai, Q. J. Niu, *Desalination* **2020**, *488*, 114525.

[30] Y. Liu, J. Zhu, J. Zheng, X. Gao, J. Wang, X. Wang, Y. F. Xie, X. Huang, B. Van der Bruggen, *Environ. Sci. Technol.* **2020**, *54*, 1946.

[31] S. Yang, Q. Jiang, K. Zhang, *J. Membr. Sci.* **2020**, *604*, 118052.

[32] R. Hu, Y. He, C. Zhang, R. Zhang, J. Li, H. Zhu, *J. Mater. Chem. A* **2017**, *5*, 25632.

[33] Y. Lin, Q. Shen, Y. Kawabata, J. Segawa, X. Cao, K. Guan, T. Istirokhatun, T. Yoshioka, H. Matsuyama, *Chem. Eng. J.* **2021**, *420*, 127602.

[34] K. Shen, C. Cheng, T. Zhang, X. Wang, *J. Membr. Sci.* **2019**, *588*, 117192.

[35] J. Zhu, S. Yuan, A. Uliana, J. Hou, J. Li, X. Li, M. Tian, Y. Chen, A. Volodin, B. Van der Bruggen, *J. Membr. Sci.* **2018**, *554*, 97.

[36] W. Shang, F. Sun, W. Jia, J. Guo, S. Yin, P. W. Wong, A. K. An, *J. Membr. Sci.* **2020**, *600*, 117852.

[37] Y. Liu, X. Wang, X. Gao, J. Zheng, J. Wang, A. Volodin, Y. F. Xie, X. Huang, B. Van der Bruggen, J. Zhu, *J. Membr. Sci.* **2020**, *596*, 117717.

[38] Z. Liao, X. Fang, J. Xie, Q. Li, D. Wang, X. Sun, L. Wang, J. Li, *ACS Appl. Mater. Interfaces* **2019**, *11*, 5344.

[39] M. B. M. Y. Ang, C.-L. Tang, M. R. De Guzman, H. L. C. Maganto, A. R. Caparanga, S.-H. Huang, H.-A. Tsai, C.-C. Hu, K.-R. Lee, J.-Y. Lai, *Desalination* **2020**, *481*, 114352.

[40] F. Yan, H. Chen, Y. Lü, Z. Lü, S. Yu, M. Liu, C. Gao, *J. Membr. Sci.* **2016**, *513*, 108.

[41] A. Bera, J. S. Trivedi, S. K. Jewrajka, P. K. Ghosh, *J. Membr. Sci.* **2016**, *519*, 64.

[42] X. Kong, Y. Zhang, S.-Y. Zeng, B.-K. Zhu, L.-P. Zhu, L.-F. Fang, H. Matsuyama, *J. Membr. Sci.* **2016**, *518*, 141.

[43] M. B. M. Y. Ang, Y.-L. Ji, S.-H. Huang, H.-A. Tsai, W.-S. Hung, C.-C. Hu, K.-R. Lee, J.-Y. Lai, *J. Membr. Sci.* **2017**, *53*9, 52.

[44] L. Bai, Y. Liu, N. Bossa, A. Ding, N. Ren, G. Li, H. Liang, M. R. Wiesner, *Environ. Sci. Technol.* **2018**, *52*, 11178.

[45] W. Fang, L. Shi, R. Wang, *J. Membr. Sci.* **2013**, *430*, 129.

[46] Z.-L. Qiu, L.-F. Fang, Y.-J. Shen, W.-H. Yu, B.-K. Zhu, C. Hélix-Nielsen, W. Zhang, *ACS Nano* **2021**, *15*, 7522.

[47] Y.-L. Ji, Q.-F. An, X.-D. Weng, W.-S. Hung, K.-R. Lee, C.-J. Gao, *J. Membr. Sci.* **2018**, *548*, 559.

[48] B.-Q. Huang, Y.-J. Tang, Z.-X. Zeng, Z.-L. Xu, *J. Membr. Sci.* **2020**, *596*, 117718.

[49] W. Fang, L. Shi, R. Wang, *J. Membr. Sci.* **2014**, *468*, 52.

[50] J. S. Trivedi, D. V. Bhalani, G. R. Bhadu, S. K. Jewrajka, *J. Mater. Chem. A* **2018**, *6*, 20242.

[51] H.-C. Yang, M.-B. Wu, J. Hou, S. B. Darling, Z.-K. Xu, *J. Mater. Chem. A* **2018**, *6*, 2908.

[52] J.-J. Wang, H.-C. Yang, M.-B. Wu, X. Zhang, Z.-K. Xu, *J. Mater. Chem. A* **2017**, *5*, 16289.

[53] Z. Sun, Q. Wu, C. Ye, W. Wang, L. Zheng, F. Dong, Z. Yi, L. Xue, C. Gao, *Nano Lett.*

2019, *19*, 2953.
[54] C. Wei, Z. He, L. Lin, Q. Cheng, K. Huang, S. Ma, L. Chen, *J. Membr. Sci.* **2018**, *563*, 752.
[55] G. Gong, P. Wang, Z. Zhou, Y. Hu, *ACS Appl. Mater. Interfaces* **2019**, *11*, 7349.
[56] Y.-J. Tang, L.-J. Wang, Z.-L. Xu, Y.-M. Wei, H. Yang, *J. Membr. Sci.* **2016**, *502*, 106.
[57] A. Akbari, E. Aliyarizadeh, S. M. M. Rostami, M. Homayoonfal, *Desalination* **2016**, *377*, 11.
[58] D. Ren, X.-T. Bi, T.-Y. Liu, X. Wang, *J. Mater. Chem. A* **2019**, *7*, 1849.
[59] Q. Shi, L. Ni, Y. Zhang, X. Feng, Q. Chang, J. Meng, *J. Mater. Chem. A* **2017**, *5*, 13610.
[60] Z. Tan, S. Chen, X. Peng, L. Zhang, C. Gao, *Science* **2018**, *360*, 518.
[61] C. Ji, S. Xue, Y.-J. Tang, X.-H. Ma, Z.-L. Xu, *ACS Appl. Polym. Mater.* **2019**, *2*, 585.
[62] L. Zhang, R. Zhang, M. Ji, Y. Lu, Y. Zhu, J. Jin, *J. Membr. Sci.* **2021**, *636*, 119478.
[63] S.-M. Xue, Z.-L. Xu, Y.-J. Tang, C.-H. Ji, *ACS Appl. Mater. Interfaces* **2016**, *8*, 19135.
[64] Z. Yao, H. Guo, Z. Yang, W. Qing, C. Y. Tang, *Desalination* **2018**, *445*, 115.
[65] J. Zhu, J. Hou, R. Zhang, S. Yuan, J. Li, M. Tian, P. Wang, Y. Zhang, A. Volodin, B. Van der Bruggen, *J. Mater. Chem. A* **2018**, *6*, 15701.
[66] K. Shen, P. Li, T. Zhang, X. Wang, *J. Membr. Sci.* **2020**, *607*, 118153.
[67] G. Kong, L. Fan, L. Zhao, Y. Feng, X. Cui, J. Pang, H. Guo, H. Sun, Z. Kang, D. Sun, *J. Membr. Sci.* **2021**, *622*, 119045.
[68] H. J. Rezania, V. Vatanpour, A. Shockravi, M. Ehsani, *Sep. Purif. Technol.* **2019**, *222*, 284.
[69] Z.-M. Zhan, Z.-L. Xu, K.-K. Zhu, S.-M. Xue, C.-H. Ji, B.-Q. Huang, C. Y. Tang, Y.-J. Tang, *J. Membr. Sci.* **2020**, *604*, 118067.
[70] H. Peng, Q. Tang, S. Tang, J. Gong, Q. Zhao, *J. Membr. Sci.* **2019**, *592*, 117386.
[71] Z. Gu, P. Li, X. Gao, Y. Qin, Y. Pan, Y. Zhu, S. Yu, Q. Xia, Y. Liu, D. Zhao, *J. Membr. Sci.* **2021**, *625*, 119144.
[72] N. Song, X. Xie, D. Chen, G. Li, H. Dong, L. Yu, L. Dong, *J. Membr. Sci.* **2021**, *621*, 118985.
[73] Y.-J. Tang, Z.-L. Xu, S.-M. Xue, Y.-M. Wei, H. Yang, *J. Membr. Sci.* **2017**, *541*, 483.
[74] Z. Yang, Z.-w. Zhou, H. Guo, Z. Yao, X.-h. Ma, X. Song, S.-P. Feng, C. Y. Tang, *Environ. Sci. Technol.* **2018**, *52*, 9341.
[75] M.-B. Wu, Y. Lv, H.-C. Yang, L.-F. Liu, X. Zhang, Z.-K. Xu, *J. Membr. Sci.* **2016**, *515*, 238.
[76] Y. Chen, F. Liu, Y. Wang, H. Lin, L. Han, *J. Membr. Sci.* **2017**, *537*, 407.
[77] H. Sun, P. Wu, *J. Membr. Sci.* **2018**, *564*, 394.
[78] M. Wu, J. Yuan, H. Wu, Y. Su, H. Yang, X. You, R. Zhang, X. He, N. A. Khan, R. Kasher, *J. Membr. Sci.* **2019**, *576*, 131.
[79] B. Yuan, C. Jiang, P. Li, H. Sun, P. Li, T. Yuan, H. Sun, Q. J. Niu, *ACS Appl. Mater. Interfaces* **2018**, *10*, 43057.
[80] Y. Lin, X. Yao, Q. Shen, T. Ueda, Y. Kawabata, J. Segawa, K. Guan, T. Istirokhatun, Q. Song, T. Yoshioka, H. Matsuyama. *Nano Lett.* **2021**, *21*, 6525.
